# Argonaute 2 drives resistance to immune checkpoint inhibitors in immunorefractory non-small cell lung cancer

**DOI:** 10.1371/journal.pbio.3003860

**Published:** 2026-06-18

**Authors:** Dario Pasquale Anobile, Layla Barbar, Emile Maucotel, Alexis Cornec, Valeria Manriquez, Wilfrid Richer, Jordan Denizeau, Christine Sedlik, Charlie Bories, Elodie Couderc, Renaud Leclere, Judith Sobas, Emeline Papillon, Rafael Mena Osuna, Jimena Tosello-Boari, Marianne Burbage, Eliane Piaggio, Enzo Z. Poirier

**Affiliations:** 1 Innate Immunity in Physiology and Cancer Team, Institut Curie, PSL Research University, INSERM U932, Paris, France; 2 Translational Immunotherapy Team, Institut Curie, PSL Research University, INSERM U932, Paris, France; 3 Department of Translational Research, PSL University, Institut Curie Research Center, Paris, France; 4 Department of Diagnostic and Theragnostic Medicine, BioHub, Pathex, Institut Curie Research Center, Paris, France; 5 Tolerance and Anti-tumour Immunity Team, Institut Curie, PSL Research University, INSERM U932, Paris, France; University of Bern, SWITZERLAND

## Abstract

One of the first-line treatments for advanced non-small cell lung cancer (NSCLC) are immune checkpoint inhibitors (ICI), which activate the antitumor immune response. Despite their success, ICI remain ineffective in many patients, highlighting the need for strategies to overcome resistance. Most efforts have focused on promoting immune cell infiltration into refractory tumors to improve ICI efficacy. In this work, we mobilize this approach by focusing on Argonaute 2 (Ago2), a pivotal member of the RNA interference pathway. Using two murine models of immunorefractory NSCLC, we demonstrate that tumoral Ago2 suppresses interferon signaling, leading to poor immunogenicity and failure of ICI therapy. Genetic deletion of Ago2 in cancer cells restores interferon signaling and supports immune infiltration of the tumor. Consequently, whereas wild-type tumors are resistant to ICI, tumors devoid of Ago2 become sensitive to treatment. In NSCLC patients treated with ICI, high Ago2 expression and a low interferon signature in tumors correlate with reduced survival. Ago2 is thus a driver of the immunorefractory phenotype observed in NSCLC and may represent a therapeutic target when aiming to sensitize patients to ICI.

## Abstract 2

Immune checkpoint inhibitors are used to treat advanced non-small cell lung cancer to activate an antitumor immune response, however they fail in many patients due to resistance. This study shows that Argonaute 2 suppresses immune infiltration in lung tumors and that its deletion sensitizes tumors to immunotherapy.

## Introduction

Lung cancer remains a leading cause of cancer-related deaths globally, with non-small cell lung cancer (NSCLC) accounting for nearly 80% of all cases [1]. For patients with advanced NSCLC, immune checkpoint inhibitors (ICI) therapy has become one of the standard first-line treatments [2]. The ICI strategy relies on targeting cell surface molecules, termed checkpoints, responsible for attenuating the immune response [3]. Cancer cells express high levels of checkpoint molecules such as PD-L1, which inhibit the antitumoral immune response, including the cytotoxic effect of CD8^+^ T cells, that are central effectors of adaptive immunity [4]. Consequently, inhibiting checkpoint molecules, via an antibody targeting PD-1, for example, unleashes the immune response against tumors. The introduction of ICI in the clinic over the past decade has represented a medical breakthrough, with groundbreaking success in several types of solid tumors [5]. Yet, clinical responses are often incomplete, and a significant number of patients experiences resistance to treatment [6,7]. There is thus a pressing need to identify therapeutic strategies that circumvent resistance to ICI [3].

The effectiveness of ICI is closely influenced by the tumor microenvironment (TME), a complex ecosystem composed of non-malignant cells such as fibroblasts and endothelial cells, along with infiltrating immune cells [8]. The immune compartment of the TME is composed of myeloid cells, such as macrophages and dendritic cells, which uptake material of tumoral origin and activate the adaptive branch of immunity, embodied by B and T cells [9,10]. CD8^+^ T cells play a central role in tumor control, as they recognize and eliminate tumor cells in an antigen-specific manner [11]. In NSCLC, the composition of the TME, particularly of the immune compartment, exhibits patient-specific heterogeneity [4]. Tumors that are highly infiltrated by immune cells, usually coined ‘hot’, contain a breadth of immune cell types embedded within the tumor mass. Immune infiltration is favored by an inflammatory TME, characterized by the presence of type I and II interferons (IFN) and pro-inflammatory cytokines of innate immunity [12]. IFN signaling results on the transcriptional upregulation of hundreds of interferon-stimulated genes (ISGs), including cytokines such as C-C motif ligand 2 (CCL2) and C-X-C motif chemokine ligand 10 (CXCL10), that contribute to attracting myeloid cells and T cells into the tumor [10,13]. Type I and II IFNs also facilitate the priming of antitumor T cell responses, particularly by promoting the activation of CD8⁺ T cells [14,15]. Hot tumors respond preferentially to ICI, as documented in NSCLC, where enrichment of PD-L1^+^ macrophages and dendritic cells is associated with greater sensitivity to anti-PD1 therapy [4,16–18]. Oppositely, a significant proportion of tumors display low immune infiltration, either due to the absence of immune cells, or because of a blockade of immune cells at the tumor periphery [4]. These so-called ‘cold’ tumors are less sensitive to ICI [5,19,20]. Consequently, strategies to enhance the efficacy of ICI have focused on remodeling the TME to promote immune cell infiltration [21]. In essence, the goal is to shift tumors from cold to hot, which can be achieved by increasing inflammation within the TME [12].

Argonaute 2 (Ago2) is a pivotal player of the RNA interference (RNAi) pathway, which regulates the expression of genes at the post-transcriptional level. Ago2 relies on micro-RNAs (miRNAs) produced by Dicer1 to target messenger RNAs and regulate their translation [22]. The mammalian Argonaute family comprises four paralogs, all of which bind microRNAs and guide them to target mRNAs. Among them, Ago2 is the only member with catalytic activity capable of cleaving target RNAs [22,23]. In addition to regulating gene expression, Ago2 and the RNAi pathway are involved in controlling the accumulation of double-stranded RNA (dsRNA) in the cytosol, which can arise from secondary structures within endogenous RNAs, as well as from the transcription of transposable elements (TEs) [24,25]. Uncontrolled accumulation of cytosolic dsRNA triggers an innate immune response, leading to the production of IFNs and pro-inflammatory cytokines [26–28]. Dysregulation of Argonaute proteins has been recently reported in various types of cancer, including NSCLC, where high expression of Ago2 correlates with reduced patient survival [29–31]. The role of Ago2 in controlling the accumulation of immunostimulatory dsRNA, as well as the association between Ago2 expression and cancer survival, suggest that Ago2 may play a part in tuning the antitumoral immune response.

## Results

### Loss of Ago2 dampens tumor growth in immunocompetent mice

**Fig 1:**
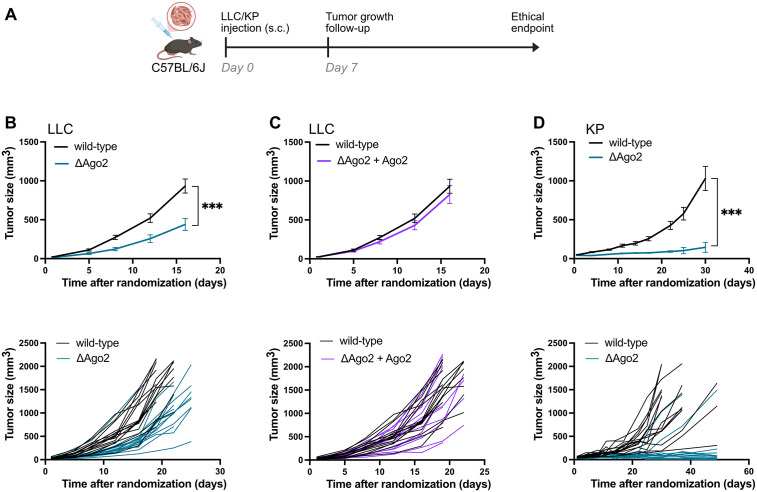
Loss of Ago2 dampens tumor growth in immunocompetent mice. **(A)** Schematics of tumor engraftment and tumor growth follow-up. *Created in BioRender. Poirier,* ***E.*** *(2026)* *https://BioRender.com/17igj4k*. **(B)** Wild-type mice were implanted with LLC wild-type or ΔAgo2 cells and tumor growth was measured with a caliper. **(C)** Wild-type mice were implanted with LLC wild-type or ΔAgo2 cells complemented for the expression of Ago2 (ΔAgo2 + Ago2) and tumor growth was measured. **(D)** Wild-type mice were implanted with KP wild-type or ΔAgo2 cells and tumor growth was measured. Mean ± SEM (top) and individual tumor growth (bottom) are represented. (B), (C), and (D) 16 mice per group, pooled from 2 independent experiments of 8 mice each. Statistical analysis was performed using two-way repeated-measures ANOVA followed by Šidák post-hoc test; *p < 0.05, **p < 0.01, and ***p < 0.001. The underlying numerical data for this figure can be found in S1 Data.

Lewis lung carcinoma (LLC) cells represent a classical syngeneic murine model of NSCLC, established by subcutaneously implanting cells on the flank of mice and monitoring tumor growth. LLC tumors are poorly infiltrated by immune cells, serving as a prototypical ‘cold’ NSCLC model, refractory to ICI treatment [32,33]. To interrogate the role of Ago2 in NSCLC, we generated Ago2-knocked out (ΔAgo2) LLC cells (S1A Fig). Deletion of Ago2 does not alter the proliferative capacity of LLC cells in vitro (S1B Fig). In wild-type syngeneic mice, lack of Ago2 in tumors translated into a 2-fold reduction in growth by day 16 with respect to wild-type tumors (Fig 1A and 1B). Complementation of Ago2 expression in ΔAgo2 tumors restored tumor growth to wild-type levels, indicating that the defect in growth is indeed attributable to the absence of Ago2 (Figs 1C and S1A). Thus, Ago2 expression favors LLC tumor growth. To investigate if the pro-tumorigenic effect of Ago2 is specific to LLC cells, we utilized KP cells, a second, orthogonal model of NSCLC. KP cells carry an oncogenic mutation in Kras (Kras^G12D^) as well as a deletion of the tumor suppressor p53. KP tumors are ‘cold’, poorly infiltrated by immune cells, and unresponsive to ICI treatment [34,35]. Compared to wild-type cells, ΔAgo2 KP cells showed a 7-fold decrease in tumor growth when implanted in wild-type mice by day 30 (Figs 1D and S1A). These data indicate that Ago2 expression promotes tumor growth in two models of NSCLC. It suggests that inhibiting Ago2 may constitute an anticancer therapeutic strategy.

To explore the therapeutic potential of targeting Ago2, we aimed to identify small-molecule inhibitors of the protein. Acriflavine (ACF) and aurintricarboxylic acid (ATA) have been documented to bind Ago2 and inhibit its activity, thereby shutting down the RNAi pathway [36–38]. We verified that ACF and ATA indeed inhibit dsRNA-driven RNAi by using a cellular fluorescent reporter system (S1C Fig). Before testing these molecules in mouse tumor models, we evaluated their toxicity through daily intraperitoneal injections (S1D Fig). ATA showed no signs of toxicity within the 3–30 mg/kg range, while ACF induced weight loss at 30 mg/kg but was well tolerated at lower doses (S1D Fig). We therefore selected the non-toxic doses of 12.5 mg/kg for ACF and 0.1 mg/kg for ATA for subsequent in vivo experiments. Wild-type mice bearing wild-type LLC tumors showed a 2-fold reduction in tumor growth upon 16 days of ACF treatment (S1E and S1F Fig). Administration of ATA translated into a 30% decrease in LLC tumor growth by day 17 (S1G Fig). Small-molecules inhibitors of Ago2 are thus antitumoral in a syngeneic NSCLC mouse model. To probe the specificity of ACF’s antitumoral effect, wild-type mice bearing ΔAgo2 LLC cells were treated with the inhibitor. In tumors lacking Ago2, implanted in Ago2-competent mice, ACF treatment did not affect tumor growth (S1H Fig). Restoring Ago2 expression in ΔAgo2 cells reinstated the antitumoral effect of the small-molecule inhibitor (S1I Fig). These findings suggest that the Ago2 inhibitors ACF and ATA exert antitumor effects by selectively targeting Ago2, primarily within tumor cells.

Altogether, these data demonstrate that Ago2 expression in NSCLC is a driver of tumor growth.

### Ago2 suppresses tumor-intrinsic interferon signaling

**Fig 2:**
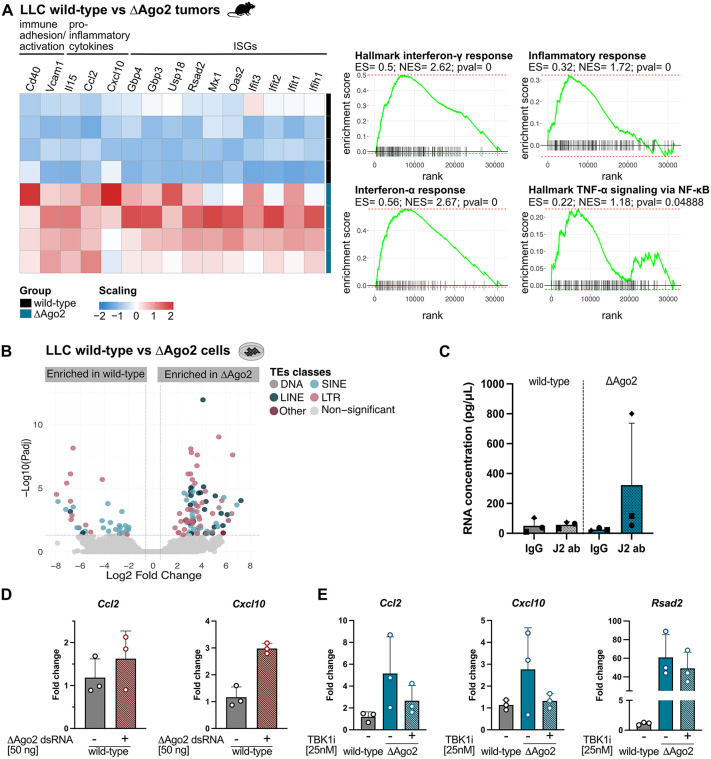
Ago2 suppresses tumor-intrinsic interferon signaling. **(A)** Curated heatmap of selected genes upregulated in tumor-derived ΔAgo2 cells relative to wild-type. Pathway enrichment analyses performed using fastGSEA, with enrichment plots shown for the four most relevant pathways identified by Gene Ontology and pathway databases. *Created in BioRender. Poirier, E. (2026) https://BioRender.com/17igj4k.* **(B)** Volcano plot of significantly differentially expressed TEs classes in ΔAgo2 vs. wild-type cells cultured in vitro (*p* < 0.05; |log_2_ fold change| > 1,2). *Created in BioRender. Poirier,* ***E.*** *(2026)* *https://BioRender.com/e898m22*. **(C)** Immunoprecipitation of dsRNA using the J2 antibody in WT and ΔAgo2 cells (*n* = 3). RNA was quantified using the Agilent 2100 Bioanalyzer with the RNA 6000 Pico chip. **(D)** Wild-type LLC cells were transfected with 50 ng of dsRNA immunoprecipitated from ΔAgo2 cells. Levels of *Ccl2* and *Cxcl10* transcripts were measured by RT-qPCR at 16 h post-transfection. **(E)** ΔAgo2 cells were exposed to the TBK1 inhibitor BX795 at 25 nM for 72 h. Levels of *Ccl2*, *Cxcl10* and *Rsad2* transcripts were measured by RT-qPCR at the end of the incubation. The underlying numerical data for this figure can be found in S1 Data.

To investigate the mechanisms driving the pro-tumoral role of Ago2, we performed transcriptomics analysis on wild-type and ΔAgo2 LLC tumors, following their isolation from syngeneic wild-type mice 12 days post-implantation. 406 genes exhibited at least a 1.2-fold change in expression between wild-type and ΔAgo2 tumors, with 394 of them upregulated in ΔAgo2 tumors (S2A Fig and S1 Table). Genes with increased expression in ΔAgo2 tumors included a panel of ISGs such as melanoma differentiation-associated protein 5 (Mda5, an innate immune receptor encoded by *Ifih1*), the antiviral effectors Mx dynamin-like GTPase 1 (Mx1) and radical S-adenosyl methionine domain containing 2 (Rsad2), the pro-inflammatory cytokines Ccl2 and Cxcl10, as well as Cd40 and vascular cell adhesion protein 1 (Vcam1), pertaining to the class of immune activation and adhesion genes (Fig 2A). Pathway enrichment analysis indicated that ΔAgo2 tumors significantly upregulated the expression of genes involved in the IFNα and IFNγ pathways, which belong to type I and type II IFNs, respectively (S2B Fig). Furthermore, to assess coordinated transcriptional changes, all genes were analyzed using Fast Gene Set Enrichment Analysis (fastGSEA). The analysis showed that gene sets related to IFNα and IFNγ, inflammatory response and TNFα signaling were significantly upregulated in the tumors lacking Ago2 (Fig 2A). Thus, loss of Ago2 in cancer cells translates into increased activation of the IFN pathway and promotes inflammation in vivo. This analysis did not allow to determine whether the increased inflammation originates specifically from cancer cells, since it was performed using whole tumors that also contain non-cancerous components of the TME. To address this, we performed a second transcriptomics analysis on ‘pure’ wild-type and ΔAgo2 LLC cells maintained in culture (S2C–S2F Fig). Similarly to tumors in vivo, ΔAgo2 LLC cells showed increased expression of ISGs such as Ifih1 and Ccl2 (S2D Fig and S1 Table). Fewer genes associated with IFN signaling and inflammation were upregulated under cell culture conditions compared to in vivo, which may explain the inconclusive results of the pathway enrichment analysis (S2E Fig). These findings suggest that, in vivo, cells within the TME contribute to the Ago2-dependent inflammatory signature alongside cancer cells.

To better understand the mechanisms underlying the inflammatory signature observed in ΔAgo2 LLC cells, we investigated the role of TEs, genomic sequences that comprise nearly half of both the mouse and human genomes [39,40]. TEs encompass diverse and unrelated families, including long interspersed nuclear elements (LINEs) and long terminal repeat (LTR)-containing elements. Their expression is typically repressed to safeguard genomic integrity and prevent transcriptional dysregulation. In addition to epigenetic silencing mechanisms such as histone modifications and DNA methylation, Ago2 and the RNAi pathway have been shown to suppress the activity of specific TE subfamilies, notably LINE1 and endogenous retrovirus K (ERVK, a type of LTR element) [39,35]. In cancer cells, elevated transcription of specific TE subfamilies can lead to the accumulation of immunostimulatory dsRNA, thereby triggering an inflammatory response [26,28,44,45]. Drawing from these data, we hypothesized that the loss of Ago2 in LLC cells may result in increased TE expression and a subsequent buildup of immunostimulatory dsRNA. To test this, we reanalyzed the bulk transcriptomic data of wild-type and ΔAgo2 LLC cells maintained in culture (Fig 2B). This revealed a significant upregulation of 93 discrete TEs in ΔAgo2 cells compared to wild-type controls (Figs 2B, S3A, and S3B; S3 Table). Notably, elements from the LINE and LTR families were overrepresented among those with increased transcription following Ago2 loss (S3B and S3C Fig). This includes members of the LINE1 and ERVK subfamilies, consistent with previous reports implicating RNAi in their transcriptional repression [41–43]. Furthermore, we observed elevated expression of TEs from the RSINE1, RLTR45, MMERVK10D3, and MMERGLN subfamilies, as well as additional ERVK elements (S3 Table). These have previously been associated with inflammatory signaling and dsRNA accumulation [46–49]. Taken together, these findings suggest that the transcriptional derepression of TEs in ΔAgo2 LLC cells may contribute to the observed inflammatory phenotype through the accumulation of immunostimulatory dsRNA [44]. To investigate this, we immunoprecipitated dsRNA from wild-type and ΔAgo2 LLC cells using the J2 monoclonal antibody, which specifically recognizes dsRNA. The precipitated RNA was then analyzed by gel electrophoresis. Compared to wild-type cells, ΔAgo2 LLC cells exhibited a mean 6-fold increase in dsRNA accumulation, indicating a substantial elevation in dsRNA levels upon Ago2 loss (Fig 2C). Accumulation of dsRNA in the cytosol, whether from endogenous or exogenous origin, is a well-established trigger of innate immune responses. We therefore assessed the immunogenic potential of dsRNA isolated from ΔAgo2 LLC cells, by transfecting it into wild-type LLC cells. dsRNA purified from ΔAgo2 LLC cells triggered an inflammatory response, marked by the induction of the cytokines *Ccl2* and *Cxcl10* (Fig 2D). This indicates that loss of Ago2 in cancer cells translates into accumulation of dsRNA which can activate inflammation. TE‑derived dsRNA is known to elicit inflammatory responses through recognition by the pattern‑recognition receptors RIG‑I and MDA5, which signal via the adaptor protein MAVS, as well as via TLR3 [28,50]. To assess the contribution of RIG‑I and MDA5 to the sensing of TE‑derived dsRNA, we knocked out *Mavs* in an Ago2‑deficient background, generating ΔAgo2,ΔMAVS LLC cells (S3D Fig). The elevated expression of inflammatory target genes observed in ΔAgo2 cells was maintained in ΔAgo2,ΔMAVS cells, indicating that signaling downstream of RIG‑I and MDA5 alone is insufficient to fully account for the inflammatory phenotype induced by Ago2 loss (S3E Fig). This may be explained by recent evidence suggesting that chronic MAVS deficiency engages compensatory inflammatory signaling through the TLR pathway [51]. Previous studies have also demonstrated that TE-derived dsRNA can be simultaneously sensed by RIG-I/MDA5 and TLR3, raising the possibility that TLR3 signaling sustains inflammation in the absence of MAVS [50]. To test this, we targeted TBK1, a shared downstream kinase in RIG-I, MDA5, and TLR3 signaling. Inhibition of TBK1 with the small molecule BX795 reduced inflammatory gene expression in ΔAgo2 LLC cells (Fig 2E). These data support a model in which loss of Ago2 leads to the accumulation of immunostimulatory, TE-derived dsRNA that is detected through convergent RIG-I/MDA5 and TLR3 signaling pathways.

Altogether, these data demonstrate that Ago2 loss induces a tumor‑intrinsic inflammatory state, including elevated expression of the chemokines CCL2 and CXCL10, which are known to promote myeloid and T cell recruitment in tumors [10,13]. This Ago2‑dependent pro‑inflammatory state may therefore have an impact on the antitumoral immune response.

### Ago2 antagonizes the antitumoral immune response

**Fig 3:**
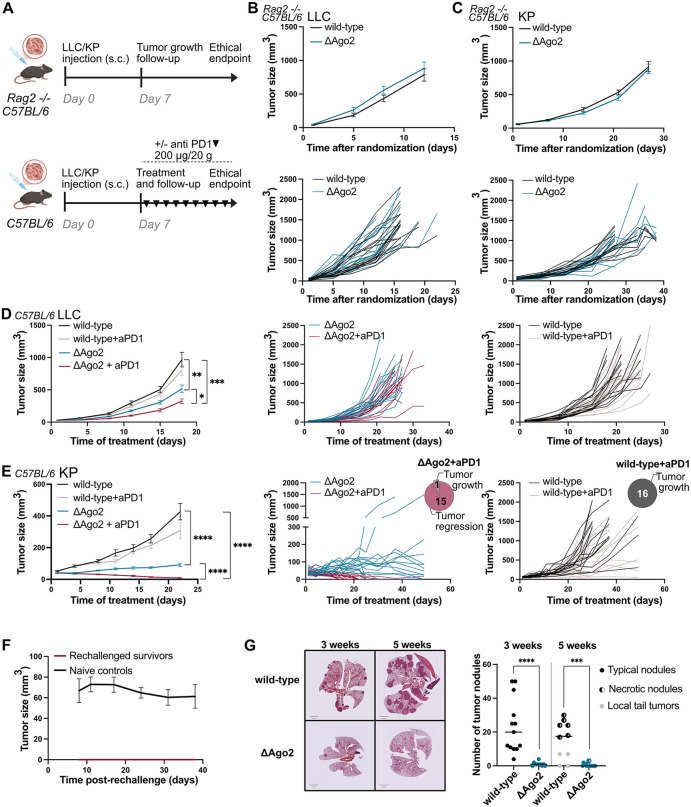
Ago2 antagonizes the antitumoral immune response. **(A)** Schematics of tumor engraftment, treatment administration, and tumor growth follow-up. *Created in BioRender. Poirier, E. (2026) https://BioRender.com/17igj4k.* **(B)** Rag2^−/−^ mice were implanted with LLC wild-type or ΔAgo2 cells and tumor growth was measured. Mean ± SEM (top) and individual tumor growth (bottom) are represented. **(C)** Rag2^−/−^ mice were implanted with KP wild-type or ΔAgo2 cells and tumor growth was measured. Mean ± SEM (top) and individual tumor growth (bottom) are represented. **(D)** Wild-type mice were implanted with LLC wild-type or ΔAgo2 cells. Anti-PD1 was administered three times a week at a dose of 200 μg/20 g and tumor growth was measured. Mean ± SEM (left) and individual tumor growth (right) are represented. **(E)** Wild-type mice were implanted with KP wild-type or ΔAgo2 cells. Anti-PD1 was administered three times a week at a dose of 200 μg/20 g and tumor growth was measured. Mean ± SEM (left) and individual tumor growth (right) are represented. (B), (D), (E) 16 mice per group, pooled from 2 independent experiments of 8 mice each. (C), one experiment of 8 mice per condition. Statistical analysis was performed using two-way repeated-measures ANOVA followed by Šidák post-hoc test; *p < 0.05, **p < 0.01, and ***p < 0.001. **(F)** 12 wild-type mice that successfully cleared initial ΔAgo2 KP tumors upon anti-PD1 treatment were rechallenged with the same cell line at 100 days post-treatment initiation. A cohort of 15 naive mice was injected in parallel as a control. **(G)** Representative hematoxylin and eosin staining of lung sections from wild-type mice at 3 and 5 weeks post-injection with either wild-type or ΔAgo2 KP cells. Microscopic quantification of tumor nodules was performed by a blinded expert pathologist. Statistical analysis was performed using Mann–Whitney; *p < 0.05, **p < 0.01, and ***p < 0.001. The underlying numerical data for this figure can be found in S1 Data.

Restoring IFN signaling in NSCLC drives immune cell recruitment in tumors, thereby favoring the antitumor immune response, and particularly the cytotoxic effect of CD8^+^ T cells [52]. Consequently, we hypothesized that the decreased growth observed for ΔAgo2 tumors could be due to an IFN-driven activation of the antitumoral immune response. To probe this notion, we interrogated the role of adaptive immunity in controlling tumor growth, by using Rag2^−/−^ mice, devoid of T and B cells. In contrast to wild-type immunocompetent mice, ΔAgo2 LLC tumors displayed similar growth to wild-type tumors in Rag2^−/−^ mice (Fig 3A and 3B). Notably, ΔAgo2 LLC tumors implanted in Rag2^−/−^ mice exhibited greater growth than wild-type tumors implanted in wild-type immunocompetent mice (S4A Fig). We next examined the contribution of adaptive immunity to the growth of KP tumors. ΔAgo2 KP tumors grew comparably to wild-type tumors in Rag2^−/−^ mice, in stark contrast to the pronounced growth delay observed in immunocompetent hosts (Fig 3C). These findings indicate that the impaired growth of ΔAgo2 tumors is imposed by the adaptive immune response.

In addition to genetic deletion of Ago2, we demonstrated that the small-molecule inhibitor ACF also exhibits antitumor activity (S1F Fig). We thus investigated whether this effect depends on the adaptive immune response. Wild-type LLC cells were implanted into Rag2^−/−^ mice, which were subsequently treated with either ACF or vehicle control (S4B Fig). ACF-treated mice displayed a 2-fold reduction in tumor growth at day 13 compared to vehicle-treated mice, which was equivalent to the reduction in growth observed in wild-type mice (S1F and S4B Figs). This indicates that ACF reduces tumor growth independently of the action of the adaptive immune system. Given that ACF may influence host immune cells expressing Ago2, we evaluated its effect on immune cell infiltration using flow cytometry. At the early treatment time point of 5 days, tumors from ACF-treated mice exhibited an approximately 2-fold reduction in total infiltrating immune cells (CD45 ⁺ compartment), a trend that persisted over time (S4C Fig). This suggests that ACF may have unintended toxic effects on immune cells. Along these lines, ACF has been reported to inhibit DNA topoisomerases [53]. To avoid confounding factors introduced by the potential off-target effects of Ago2 inhibitors, we focused hereafter on the genetic deletion of Ago2 in tumor cells.

It is well established that ICI therapy is largely ineffective at suppressing growth of wild-type LLC tumors in immunocompetent mice, a limitation attributed to poor immune cell infiltration within the TME [54]. Our data indicate that, unlike wild-type cells, ΔAgo2 LLC cells are effectively targeted by the adaptive immune system (Figs 1B and 3B). We therefore examined if Ago2 loss in LLC tumors could restore responsiveness to ICI. Growth of wild-type and ΔAgo2 LLC tumors was monitored in immunocompetent mice treated with an antibody targeting PD1 (anti-PD1, Fig 3D). As previously documented, anti-PD1 treatment did not significantly affect the growth of wild-type LLC tumors (Fig 3D). In contrast, anti-PD1-treated ΔAgo2 tumors showed a 40% decrease in tumor growth at day 18, compared to vehicle-treated ΔAgo2 tumors (Fig 3D). ΔAgo2 tumors treated with ICI showed a 3.5-fold decrease in growth at day 18 compared to wild-type tumors (Fig 3D). This indicates that loss of Ago2 sensitizes LLC tumors to ICI. We next interrogated if this effect extends beyond LLC tumors, by performing a similar experiment in KP tumor-bearing mice (Fig 3E). Consistent with the immunologically ‘cold’ nature of the KP model, anti-PD1 treatment of wild-type tumors only marginally affected tumor growth (Fig 3E) [34,36]. By contrast, ΔAgo2 tumors exhibited a striking response, with treatment resulting in near-complete growth control, and complete tumor regression in 15 out of 16 cases (Fig 3E). To determine whether tumor rejection resulted in durable protective immunity, we rechallenged mice with ΔAgo2 tumors 100 days after complete tumor clearance. Whereas naive mice readily developed tumors, mice that had previously cleared tumors were completely refractory to re‑implantation (Fig 3F). These findings indicate the establishment of immune memory following implantation with immunogenic Ago2‑deficient tumors treated with ICI.

We further evaluated whether the efficacy of ICI could be potentiated by pharmacological inhibition of Ago2, rather than genetic ablation. Because we found that ACF and ATA exhibited off‑target effects in vivo, we instead evaluated the commercial inhibitor BCI‑137 [55]. Administration of BCI-137 as a single agent yielded no significant therapeutic effect (S5 Fig). When combined with anti-PD-1, it reduced tumor burden by one-third, and extended overall survival, compared to mock and single-agent-treated tumors (S5 Fig). Consistent with our observations in Ago2-deficient tumors, these data indicate that pharmacological inhibition of Ago2 has the potential to synergize with ICI therapy. However, the magnitude of the effect is markedly reduced compared with genetic ablation of Ago2. We interpret this discrepancy as reflecting tissue-dependent activity of BCI-137, together with its likely suboptimal efficacy in immunologically ‘cold’ tumors relative to ‘hot’ tumors.

Finally, we examined the role of Ago2 in an orthotopic model of lung cancer. Following tail‑vein injection, KP cells home to the lung, their tissue of origin, and give rise to multiple tumors (S6 Fig). Tumor burden was assessed at 3 and 5 weeks post‑injection by hematoxylin and eosin staining of lung sections. In stark contrast to wild‑type KP cells, which formed an average of 15–20 tumors per animal, the vast majority of mice injected with ΔAgo2 KP cells exhibited no detectable tumors (Figs 3G and S6). These data demonstrate that Ago2 also promotes tumor growth in the lung within its native microenvironment.

These results support a role for tumoral Ago2 in suppressing antitumor immunity, limiting the efficacy of ICI treatment. Loss of Ago2 not only bolsters antitumoral immune responses but also sensitizes otherwise unresponsive NSCLC tumors to immune checkpoint inhibition.

### Ago2 loss reshapes the tumor microenvironment

**Fig 4:**
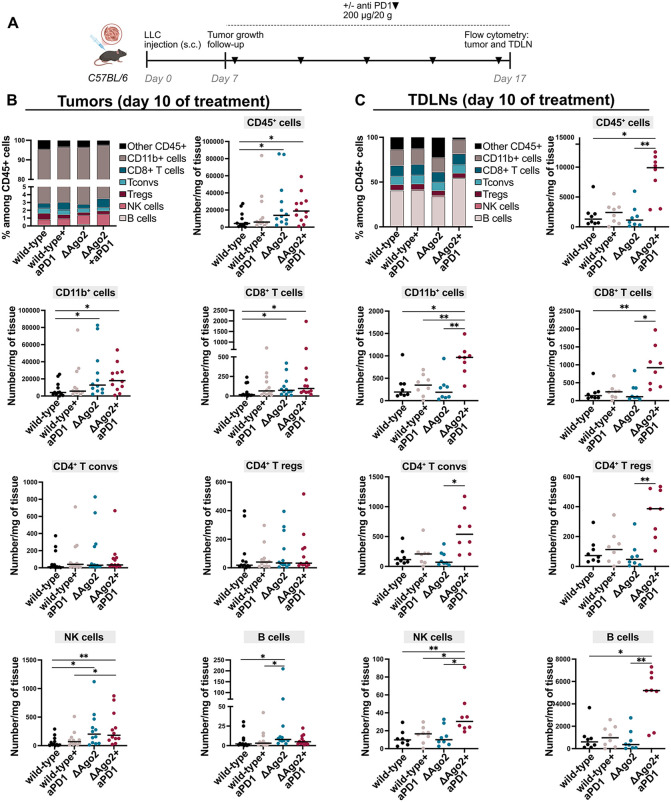
Ago2 loss reshapes the tumor microenvironment. **(A)** Schematics of tumor engraftment, treatment administration, and tumor growth follow-up. *Created in BioRender. Poirier,* ***E.*** *(2026)* *https://BioRender.com/17igj4k*. **(B)** Quantification of immune cell populations in the tumor microenvironment among CD45⁺ cells (top left). Absolute numbers of CD45⁺, CD11b⁺, CD8⁺ T cells, Tconvs, Tregs, NK cells, B cells in tumors collected 10 days after initiation of anti-PD1 treatment. Pool of 2 independent experiments. **(C)** Quantification of immune cell populations in the tumor-draining lymph nodes (TDLN) among CD45⁺ cells (top left). Absolute numbers of CD45⁺, CD11b⁺, CD8⁺ T cells, Tconvs, Tregs, NK cells, B cells in TDLNs collected 10 days after initiation of anti-PD1 treatment. One experiment. Statistical analysis was performed using Kruskal–Wallis test; *p < 0.05, **p < 0.01, and ***p < 0.001. The underlying numerical data for this figure can be found in S1 Data.

To investigate the immune alterations that confer sensitivity to ICI in Ago2-deficient tumors, we performed flow cytometry analysis on immune cells isolated from tumors and tumor-draining lymph nodes (TDLNs) of mice bearing wild-type or ΔAgo2 LLC tumors, with or without anti-PD1 treatment. Samples were collected 10 days after initiating anti-PD1 therapy, a time point at which tumor growth begins to diverge between wild-type and ΔAgo2 groups due to immune system activity (Fig 4A). Compared to wild-type untreated tumors, the total number of immune cells (CD45⁺ compartment) showed a 3-fold increase in ΔAgo2 tumors and a 4-fold increase in ΔAgo2 tumors treated with anti-PD1 (Figs 4B and S7A). Loss of Ago2 in tumors enhances immune cell infiltration, a process further amplified by ICI treatment. This increased infiltration is primarily driven by the CD11b⁺ myeloid compartment, which exhibited a 4-fold enrichment in ΔAgo2 tumors treated with anti-PD-1 compared to untreated wild-type tumors (Fig 4B). Adaptive immunity, particularly CD8⁺ T cells, plays a critical role in controlling tumor growth in LLC and other syngeneic cancer models. Our findings further demonstrate that Ago2-deficient tumors are actively targeted by the adaptive immune response (Fig 3). Consistent with these findings, ΔAgo2 tumors treated with anti-PD1 displayed an approximately 5-fold increase in CD8⁺ T cells (Fig 4B). Across all groups, intratumoral CD8⁺ T cells were predominantly of the effector phenotype, with minimal representation of naive and memory subsets (S7B Fig). Progenitor exhausted T (Tpex) cells represent a subset of PD1^+^ CD8^+^ T cells that can self-renew, thereby maintaining a pool of functional T cells that can respond to ICI therapy and differentiate into other terminally exhausted T (Tex) cell subsets [56]. ΔAgo2 tumors showed a tendency toward increased frequencies of Tpex cells, while mice bearing ΔAgo2 tumors treated with anti-PD1 exhibited the highest proportions of Tex cells (S7B Fig). The loss of Ago2 supports the maintenance of Tpex cells while also promoting their differentiation into effector-exhausted cells following ICI treatment, thereby enhancing antitumor immunity. In contrast to CD8^+^ T cells, numbers of conventional CD4⁺ T (Tconvs) cells and regulatory CD4⁺ T (Tregs) cells remained steady between conditions (Fig 4B). Natural killer (NK) cells, which recognize and directly kill tumor cells, showed an approximately 7-fold increase in ΔAgo2 tumors treated with anti-PD1, compared to wild-type untreated tumors (Fig 4B). B cells, responsible for antibody production and antigen presentation, are also present in the tumor microenvironment. Loss of Ago2 in tumors leads to a 3-fold increase in B cells (Fig 4B). Collectively, these findings show that the loss of Ago2, combined with ICI treatment, promotes immune infiltration into ‘cold’ NSCLC tumors, thereby enabling the adaptive immune system to target cancer cells and suppress tumor growth.

The antitumor immune response depends on the priming of T cells by myeloid cells within the TDLN, leading to the generation of tumor-specific T cells [10]. In the TDLN, mice bearing ΔAgo2 tumors and treated with anti-PD1 exhibited a 10-fold increase in total immune cells compared to wild-type, untreated tumors (Fig 4C). All major immune subsets contributed to this increase, including CD11b⁺ myeloid cells, CD8⁺ T cells, CD4⁺ Tconv cells, Treg cells, NK cells, and B cells (Fig 4C). In the TDLNs, anti-PD1 treatment in ΔAgo2-bearing mice tended to shift the CD8⁺ T cell population from a memory toward an effector phenotype, consistent with checkpoint-induced activation and differentiation. Nevertheless, PD1⁺ CD8⁺ cells in TDLNs predominantly retained the Tpex phenotype, with minimal representation of Tex cells across all groups. These findings support a model in which TDLNs serve as a reservoir of progenitor cells, while terminal differentiation primarily occurs within the tumor microenvironment (S8A and S8B Fig). These changes were specific to ΔAgo2 tumors treated with anti-PD1, and were absent in untreated mice, indicating a synergistic effect between Ago2 loss and ICI in promoting immune activation in TDLNs.

Altogether, these results show that Ago2 loss enhances the immune response in both tumors and TDLNs, transforming immunologically ‘cold’ tumors into an environment permissive to ICI treatment.

### Correlation between survival of ICI-treated NSCLC patients, Ago2 expression, and an IFN signature

To investigate whether Ago2-driven immunosuppression occurs in humans, we analyzed a published dataset of NSCLC patients that includes tumor transcriptomic profiles alongside survival data [57]. Patients in this cohort were all treated with ICI, specifically with anti-PD1 or anti-PD-L1. First, we evaluated whether Ago2 expression in tumor biopsies correlates with patient survival. Patients with tumors exhibiting low Ago2 levels demonstrated significantly improved survival compared to those with high Ago2 expression (S9A and S9C Fig). This observation aligns with previous reports indicating that elevated Ago2 expression in tumor biopsies is associated with poorer survival across various cancer types [31]. Next, we investigated whether the expression of inflammatory genes regulated by Ago2 correlates with patient survival. To this end, we developed an Ago2-dependent gene signature comprising genes whose expression was upregulated in ΔAgo2 tumors relative to wild-type tumors, based on our transcriptomic analysis (Fig 2A and S2 Table). Notably, Ago2 expression levels in tumors do not correlate with the Ago2-dependent gene signature (S9D Fig). This discrepancy may be due to the confounding influence of Ago2 expression, which is not limited to cancer cells but is also present in other components of the TME, as previously noted. In patient samples, elevated expression of the Ago2-dependent gene signature is associated with improved survival (S9B Fig). Since this signature encompasses ISGs such as Rsad2 and Mx1, the findings are consistent with prior studies demonstrating that patient survival is positively associated with a type I IFN signature across multiple cancer types [58,59].

Taken together, these findings suggest that, as observed in mice, tumoral Ago2 may suppress the activation of anticancer immune responses in humans, thereby reducing the efficacy of ICI. Consequently, Ago2 could represent a promising therapeutic target to enhance ICI sensitivity in NSCLC patients.

## Discussion

With this work, we demonstrate that Ago2 expression in NSCLC dampens tumor-intrinsic inflammation, thereby restraining antitumoral immune responses and the efficacy of ICI treatment. During the course of our research, Wang and colleagues published a study documenting a similar phenomenon [55]. Using mouse models of bladder cancer, colon cancer, and melanoma, which are immunologically ‘hot’ tumors that are normally responsive to ICI, the authors demonstrate that Ago2 blunts the antitumoral immune response through RNAi-mediated modulation of IFN signaling. They further show that genetic deletion of Ago2 in these tumors, as well as pharmacological inhibition of Ago2 in vivo, leads to increased antitumoral immune responses, and improves efficacy of ICI. Our work confirms and extends these findings by demonstrating that Ago2 similarly suppresses IFN signaling in immunologically ‘cold’ NSCLC tumors, which are otherwise resistant to ICI. Importantly, we show that Ago2 inhibition not only has the potential to enhance responses in tumors already sensitive to ICI but also represents a strategy to sensitize previously unresponsive tumors, broadening the therapeutic applicability of immune checkpoint blockade in NSCLC.

Our work, as well as the results of Wang *and colleagues,* point towards a central role of the IFN pathway in Ago2-mediated resistance to antitumor immunity. The two studies, however, diverge regarding the molecular mechanism by which Ago2 restrains IFN signaling. In the model by Wang and colleagues, type II IFN signaling is driven in tumor cells by IFNγ produced by T cells infiltrating the tumor, which signals via the IFNγ receptor. Ago2 is involved in reducing the levels of Stat1, that plays a central role in the IFNγ receptor signaling cascade. In this framework, IFN signaling is tumor-extrinsic, as it depends on IFNγ produced by T cells. We instead document the existence of tumor-intrinsic IFN signaling, in the absence of immune cells, as demonstrated by transcriptomics analysis of NSCLC cells in culture (Fig 2). Such differences may be related to the use of unrelated tumor models, as well as to different TMEs, particularly the immunologically ‘hot’ or ‘cold’ status. Nonetheless, it suggests that Ago2 expression is positively selected in tumor cells for its ability to counteract IFN signaling, and therefore immune activation. This inhibitory capacity may be achieved via multiple, orthogonal strategies, linked to the pleiotropic roles of Ago2. In that line, our data point to a role for Ago2 in limiting the accumulation of immunogenic, TE‑derived dsRNA. In the absence of Ago2, dsRNA accumulates and activates an innate immune response, likely through recognition by RIG‑I/MDA5 and TLR3, as previously reported [50].

Evidence links elevated Ago2 expression in tumors with reduced patient survival across different cancer types [30,31,60,61]. In hepatocellular carcinoma, Ago2 upregulation has been shown to enhance tumor cell proliferation, migration, and metastatic potential in vivo [62]. Its expression positively correlates with vascular endothelial growth factor levels across multiple hepatocellular carcinoma cell lines [63]. Similarly, in multiple myeloma, Ago2 contributes to neovascularization through microRNA-dependent mechanisms [64]. In breast cancer, Ago2 promotes tumor growth by increasing the transcription of progesterone receptor mRNA [65]. Finally, Ago2 has been shown to sustain tumor progression in Kras-driven murine models of NSCLC by interacting with Kras and promoting downstream oncogenic signaling [66,67]. Thus, Ago2 seems to possess multiple roles when it comes to favoring tumorigenesis. Whether the immunomodulatory role of Ago2 is a general feature across tumor types remains to be established.

This work demonstrates that loss of Ago2 in NSCLC cells can sensitize tumors that are otherwise resistant to ICI, highlighting Ago2 as a promising therapeutic target to enhance the efficacy of immune checkpoint blockade in patients. Treatment with two small-molecule inhibitors of Ago2, ACF, and ATA, indeed results into decreased tumor growth in vivo. However, at odds with data obtained through genetic deletion of Ago2, this effect is independent of the adaptive immune system. We hypothesize that ACF and ATA may exert off-target effects, possibly by modulating proteins such as DNA topoisomerase [53]. This modulation could influence the host immune response, potentially explaining the reduced immune infiltration observed in tumors from ACF-treated animals (S4C Fig).

We additionally evaluated BCI‑137, a reported Ago2 inhibitor. Wang and colleagues previously described a strong synergistic effect between BCI‑137 and ICI. In our study, we likewise observed a benefit of the combination treatment relative to single-agent therapies. Indeed, whereas the monotherapies were not effective, the combination of BCI and anti-PD1 resulted in a modest reduction of tumor volume and an extension of the overall survival (S5 Fig). This discrepancy may reflect differences in tumor context, including tumor type and immune microenvironment: our analyses focus on immunologically ‘cold’ tumors, whereas Wang and colleagues investigated ‘hot’ tumors. More broadly, these findings also underscore the current limitations of available Ago2 inhibitors for in vivo applications. Therefore, therapeutic targeting of Ago2 will necessitate the development of highly specific inhibitors, along with delivery strategies that ensure selective targeting of tumor cells, in order to minimize off-target effects on host cells.

## Materials and methods

### Cell lines

LLC wild-type, LLC ΔAgo2, LLC ΔAgo2, ΔMAVS, KP (Kras^LSL-G12D/+^; Trp53^flox/flox^) wild-type, and KP ΔAgo2 cells were cultured in Dulbecco’s Modified Eagle Medium (DMEM) + GlutaMAX-TM-l, 10% Embryonic Stem Cell fetal bovine serum (ESC FBS) (Gibco), and 1% penicillin-streptomycin (Gibco). HEK cells were cultured in DMEM+ GlutaMAX-TM-l + 10% FBS + 1% penicillin-streptomycin + 1% NEAA + 1% HEPES + 1% Sodium Pyruvate.

### Animal experimentation

Six to eight weeks old C57BL/6J female mice were purchased from Charles Rivers Laboratories. Mice were allowed to acclimate to the housing animal facility for 7 days before tumors engraftment. Rag2-/- mice were bred in the Animal facility of the Curie Institute. For the in vivo studies, all animals were used according to the protocols approved by the Animal Committee of Curie Institute and housed in the animal facility of the Curie Institute in a pathogen-free environment with a 12-hour day/night cycle and a free access to food and water, temperature between 20 and 24 °C with an average humidity rate between 40% and 70%. Human endpoints were used for tumor-bearing mice as maximal ethical size of tumors subcutaneously grafted of 2 cm^3^, more than 20% of weight loss, signs of altered mobility-eating ability, and cachexia. Experimental procedures were approved by the Ministère de l’Enseignement Supérieur, de la Recherche et de l’Innovation in compliance with the international guidelines. Reference APAFIS number #37693−2022061520027 172 v1.

### Tumor implantation

LLC/KP wild-type and ΔAgo2 cells were subcutaneously injected into female mice flanks (3 × 10^5^ cells in 100 μL of PBS per mouse). Mice were randomized seven days after tumor inoculation. Tumors were monitored twice a week. Tumor sizes were measured using a digital caliper and tumor volume was calculated with the formula ((width^2^ × length)/2).

Two hundreds of micrograms of anti-PD1 (Leinco Technologies) diluted in 200 μL of PBS or 200 μL of PBS were intraperitoneally injected into mice three times per week. 100 mg/kg per mouse of BCI-137 (MedChemExpress), was diluted in 10% DMSO+ 90% Corn Oil and injected intraperitoneally twice per week for the first week and once per week for the following two weeks. Treatments were initiated seven days following tumors implantation.

Induction of orthotopic lung metastases was achieved by tail vein injection of 3.5 × 10^5^ KP wild-type and ΔAgo2 cells in 200 μL of PBS.

### Flow cytometry

Mice were sacrificed 10 days after the start of the treatment and tumors and draining lymph nodes were collected. Tumors were first collected and mechanically digested in CO_2_-independent medium (Gibco) containing 0.1 mg/mL liberase TL (Roche) and 0.1 mg/mL DNAse then using gentleMACSTM Octo Dissociator (Miltenyi Biotec) for 41 min. Cell suspensions were then filtered with a 100 µm cell strainer and red blood cells were lysed with hypotonic buffer. FACS buffer (PBS 1×, 5% BSA, and 0.5 M EDTA) was then added and cells were centrifuged then Live/dead cell discrimination was performed using Live/dead fixable Aqua Dead Cell Stain Kit (Life Technologies) and Fc receptors were blocked with the CD16/CD32 (clone 2.4.G2) mAb (BD) as per the manufacturer’s instructions. Cell surface labeling was then performed by incubating cells with the antibodies listed in S4 Table diluted in FACS buffer for 30 min at 4 °C. For intracellular staining, cells were resuspended in 100 µL of fixation-permeabilization buffer (eBioscience) per sample for 30 min at 4 °C. After centrifugation, cells were incubated with the antibodies diluted in the permeabilization buffer (eBioscience) for 30 min at 4 °C. For T cell enrichment, mononuclear cells were recovered from Percoll gradient (GE Healthcare Life Science) from 40% to 75% interface, washed, and resuspended in FACS buffer before proceeding to the staining. Lymph nodes were collected in cold PBS 1×, mechanically digested, filtered, and kept in FACS buffer. Cells were stained following the same protocol as tumors. Unless specified otherwise, all centrifugations were carried out at 1,350 rpm for 8 min at 4 °C. All data acquisition was done using an LSRFortessa instrument with FACSDiva software v8.0.1 (BD) and analyzed using FlowJo software (TreeStar).

### Bulk RNA sequencing

LLC wild-type and LLC ΔAgo2 cell lines were maintained in culture as previously mentioned. RNA was purified using NucleoSpin RNA kit (MACHEREY-NAGEL) following the manufacturer’s protocol. Total RNA quality was analyzed using the Agilent 2100 Bioanalyzer G2939A. RNA libraries were prepared from 500 ng purified total RNA according to the manufacturer’s instructions. Libraries were sequenced by Illumina NovaSeq-S1-PE100, and data was aligned on a ribosomal RNAs database and then on the reference genome mm10. Gene expression is quantified using featureCounts from the software package Subread. Downstream analysis is performed using R (version 4.1.1) in which raw counts are loaded and then normalized using R package DESeq2 (version 1.34.0). Statistical analysis to identify the differential expression genes is performed using DESeq2. Genes are considered significantly differentially expressed according to Benjamini–Hochberg adjusted *p*-value. Plots are created using ggplot2 (version 3.3.6). Enrichment of gene sets was analyzed using the MSigDB gene set database available at EnrichR.

### Transposable element analysis from mouse bulk RNA-seq

Analyses were done as described in [68]. STAR (version 2.7.6.a [69]) was used to map reads against mm10 reference mouse genome. TE expressions were quantified using featureCounts [70] from Subread suite package (version 2.0.6) with the following parameters: -p -M --primary –ignoreDup for individual TE counts. The annotation file used for TE quantification was from TEtranscripts and is provided as supplementary material.

The TE count matrix was imported into R (version 4.4.2). TEs were retained for analysis only if at least one read was detected in at least two out of four replicates per group. TEs with expression below a threshold of 0.5 TPM (Transcripts Per Million) were filtered out from the raw count matrices. Counts were normalized, and differential expression analysis was conducted using the DESeq2 R package (version 1.46.0). TEs with a Benjamini–Hochberg (BH)-adjusted *p*-value <0.05 were considered significantly differentially expressed between conditions. PCA plots were generated using the plot.PCA function from the DESeq2 R package (version 1.46.0) and visualized using the ggplot function from the ggplot2 R package (version 3.5.2). Volcano plots, pie charts, and enrichment plots were created using R (version 4.4.2) and ggplot2 (version 3.5.2).

### Lentiviral CRISPR-Cas9-mediated knock out of Ago2

HEK cells were transduced with the packaging plasmid psPAX2 (Addgene), the VSV-G envelope expressing plasmid pMD2.G (Addgene), and the LentiCRISPRv2_Ago2_g2 was built according to the protocol described in [71] by cloning the Ago2_g2 sequence (GATACCTGTTCACTCTCCGA) into the backbone, to produce the lentivectors. LLC and KP cells were infected by the generated lentivirus and maintained under puromycin selection (5 µg/mL) for one week. MAVS knock-out was generated with lentivectors encoding for the plasmid VB251112-1266ncr (VectorBuilder). Cells were maintained under blasticidin selection (2 µg/mL) for 5 days. Single cells were sorted using SH800S Cell Sorter to obtain single-cells clones.

### Western blot

Cell lysis was performed by incubating cells with 25 µL of RIPA buffer (150 mM sodium chloride, 1% Triton X-100, 0.5% sodium deoxycholate, 0.1% sodium dodecyl sulfate, and 50 mM Tris, pH 8) and protease inhibitor (1/100) for 20 min on ice. Post-nuclear supernatant was mixed with 2× Laemmli Buffer containing βmercaptoethanol and boiled at 95 °C for 5 min. Samples were run in mini-PROTEAN TGX stain-free gel 4%–15% (Bio-Rad) at 90 V for 15 min then at 120 V until run was finished. The proteins were transferred on a PVDF membrane (Bio-Rad) using Trans-Blot Turbo (Bio-Rad). The membrane was blocked in 0.1% TBS-Tween and 5% milk for 1 hour at RT. Probing of the membrane was performed overnight at 4 °C with the antibody targeting Ago2 (97 KDa) and MAVS (75, 52 KDa). Membranes were then washed three times with 0.1% TBS-Tween, incubated 1 h at room temperature with HRP-coupled anti-rabbit secondary antibody and anti-beta actin antibody (42 KDa) coupled to HRP and washed again three times with 0.1% TBS-Tween. Signal detection was performed using Clarity Western ECL Substrate (Bio-Rad) on a ChemiDoc Go system (Bio-Rad). Images were acquired using either the auto-optimal exposure setting or manual signal accumulation mode (10s capture intervals over a 3min total exposure). The following antibodies were used: primary antibody: α-Ago2 (rabbit) (Cell Signaling technology, C3C4C6 rabbit mAb #2897, 1:1,000 in TBS-Tween 0.1% + milk 5%); α-MAVS (rabbit) (Cell Signaling technology, rabbit mAb #4983, 1:1,000 in TBS-Tween 0.1% + milk 5%); α-actin (Invitrogen, MA5-15739-HRP, rat mAb, 1:10,000 in TBS-Tween 0.1% + milk 1%). Secondary antibody: α-rabbit (Jackson Immuno reference #111-035-144 1:10,000 in TBS-Tween 0.1% + 1% Milk)

### Double-stranded RNA immunoprecipitation

Immunoprecipitation of dsRNA was performed using the Pierce Classic Magnetic IP Kit (Thermo Fisher) according to [72] with minor modifications. Briefly, ~8 × 10^6^ LLC cells were lysed in 500 μL lysis buffer on ice. Ten percent of each lysate was saved as input control. The remaining material was divided and incubated for overnight at 4 °C in rotation with pre-washed and pre-blocked Protein A/G magnetic beads and either an isotype control antibody (Invitrogen, Mouse IgG2a kappa Isotype Control cat #14-4724-85) or the anti-dsRNA antibody J2 (Absolute Antibody, cat. #AB01299123.0, lot #T2309A07). The following day, beads were washed three times in lysis buffer and then immediately processed for RNA extraction.

### Statistical analysis

Statistical details are indicated in the corresponding figure legends. Data were analyzed in GraphPad Prism 10 software. In figures, *p < 0.05, **p < 0.01, ***p < 0.001, and ****p < 0.0001.

## Supporting information

S1 Fig Small molecule inhibitors of Ago2 are antitumoral in NSCLC. (**A**) Western blot quantifying Ago2 protein in LLC wild-type, LLC ΔAgo2 cells, KP ΔAgo2 cells. Clone #D11.2 was chosen for in vivo engraftment. (**B**) Growth rate of wild-type and ΔAgo2 LLC and KP cells, measured by percent confluency using a CellCyte live-cell imaging system (*n* = 3). (**C**) HEK293T cells expressing GFP were transfected with dsRNA targeting part of the GFP sequence, triggering RNAi-dependent GFP silencing. Cells were treated with increasing concentrations of ACF or ATA, and GFP fluorescence was quantified by flow cytometry (*n* = 3). (**D**) Body weight curves as a measure of toxicity in mice receiving ACF (3–30 mg/kg) or ATA (3–30 mg/kg), delivered intraperitoneally, 5 days per week. Four mice per group. (**E**) Schematics of tumor engraftment, treatment administration, and tumor growth follow-up. *Created in BioRender. Poirier, E. (2026) https://BioRender.com/17igj4k* (**F**) Wild-type mice were implanted with LLC wild-type and treated with ACF 12.5 mg/kg intraperitoneally for five days/week. Tumor growth was measured. (**G**) Wild-type mice were implanted with LLC wild-type and treated with ATA 0.1 mg/kg intraperitoneally for five days/week. Tumor growth was measured. (**H**) Wild-type mice were implanted with LLC ΔAgo2 and treated with ACF 12.5 mg/kg intraperitoneally for five days/week. Tumor growth was measured. (**I**) Wild-type mice were implanted with LLC ΔAgo2 + Ago2 and treated with ACF 12.5 mg/kg intraperitoneally for five days/week. Tumor growth was measured. Mean ± SEM (left) and individual tumor growth (right) are represented. (F) and (H), 24 mice per group, pooled from 3 independent experiments of 8 mice each. (G) 11 mice per group, pooled from 2 independent experiments of 5–6 mice each. (I) 14 mice per group, pooled from 2 independent experiments of 6–8 mice each. Statistical analysis was performed using two-way repeated-measures ANOVA followed by Šidák post-hoc test; *p < 0.05, **p < 0.01, and ***p < 0.001. The underlying numerical data for this figure can be found in S1 Data.
(TIFF)

S2 Fig Transcriptomic landscape of ΔAgo2 LLC cells. (**A**) Volcano plot showing significantly differentially expressed genes in tumor-derived ΔAgo2 cells compared with tumor-derived wild-type cells (*p* < 0.05; |log_2_ fold change| > 1,2). (**B**) Pathway enrichment analysis of significantly upregulated genes in ΔAgo2 tumor-derived cells. Data were analyzed via EnrichR. (**C**) Volcano plot showing significantly differentially expressed genes in ΔAgo2 versus wild-type cells cultured in vitro (*p* < 0.05; |log_2_ fold change| > 1.2). (**D**) Curated heatmap of selected genes upregulated in ΔAgo2 cells in vitro. (**E**) Pathway enrichment analysis of significantly upregulated genes in ΔAgo2 cells cultured in vitro. Data were analyzed via EnrichR and fastGSEA, with enrichment plots shown for the four most relevant pathways identified by Gene Ontology and pathway databases. (**F**) Pathway enrichment analysis of significantly downregulated genes in ΔAgo2 cells cultured in vitro. The underlying numerical data for this figure can be found in S1 Data.
(TIFF)

S3 Fig Characterization of transposable element expression and assessment of MAVS signaling in Ago2-deficient cells. (**A**) Principal component analysis (PCA) of TE expression profiles in LLC wild-type and ΔAgo2 cells. (**B**) Relative enrichment of TE classes in wild-type and ΔAgo2 cells. (**C**) Distribution of expressed TE classes, showing the proportion of all TEs, upregulated TEs, and downregulated TEs in ΔAgo2 cells (left to right). (**D**) Western blot quantifying MAVS protein in LLC wild-type, LLC ΔAgo2 cells, LLC ΔAgo2, ΔMAVS cells. (**E**) Levels of *Ccl2*, *Cxcl10*, and *Rsad2* transcripts measured by RT-qPCR in LLC ΔAgo2 cells and LLC ΔAgo2, ΔMAVS cells. The underlying numerical data for this figure can be found in S1 Data.
(TIFF)

S4 Fig The antitumoral effect of the Ago2 inhibitor ACF is independent of the immune system. (**A**) Tumor growth of wild-type LLC engrafted in immunocompetent C57BL/6 mice and ΔAgo2 engrafted in Rag2^−/−^ mice. Mean ± SEM (left) and individual tumor growth (right) are represented. (**B**) Schematics of tumor engraftment, treatment administration, and tumor growth follow-up. Rag2^−/−^ mice were implanted with LLC wild-type and treated with ACF 12.5 mg/kg intraperitoneally for five days/week. Tumor growth was measured. 8 mice per group. One experiment. *Created in BioRender. Poirier, E. (2026) https://BioRender.com/17igj4k*. (**C**) Flow cytometry quantification of CD45^+^ immune cells in tumors treated with ACF 12.5 mg/kg intraperitoneally for five days/week and harvested at the indicated time points. Statistical analysis was performed using Mann–Whitney test; *p < 0.05, **p < 0.01, and ***p < 0.001. The underlying numerical data for this figure can be found in S1 Data.
(TIFF)

S5 Fig Pharmacological inhibition of Ago2 synergizes with ICI for a modest antitumoral effect. (**A**) Wild-type mice were implanted subcutaneously with KP wild-type and ΔAgo2 cells. Mice bearing wild-type tumors were treated with anti-PD-1 (200 µg/20 g body weight, injected intraperitoneally three times per week), BCI-137 (100 mg/kg, twice per week for the first week, then once per week for the following two weeks), or a combination of both. Mean ± SEM (left) and individual tumor growth (right) are represented. 7–8 mice per group. One experiment. Tumor sizes at day 23 post-randomization are shown. Statistical analysis was performed using Kruskall–Wallis test; *p < 0.05, **p < 0.01, and ***p < 0.001. (**B**) Kaplan–Meier survival curves for mice bearing wild-type tumors treated with vehicle, anti-PD1, BCI-137, or the combination, compared to mice bearing ΔAgo2 tumors. Statistical significance was evaluated using a log-rank test (*p* < 0.05). The underlying numerical data for this figure can be found in S1 Data.
(TIFF)

S6 Fig Loss of Ago2 dampens tumor growth in an orthotopic lung model. Representative macroscopic images of formalin-fixed lungs excised 5 weeks post-tail vein injection with either wild-type or ΔAgo2 KP cells.
(TIFF)

S7 Fig Gating strategy and immune profiling of Ago2 competent and deficient tumors. (**A**) Representative gating strategy for one tumor sample from LLC tumor-bearing mice, collected 10 days after initiation of anti-PD1 treatment. (**B**) Flow cytometry plots showing the distribution of naive, memory, and effector CD8⁺ T cells (left), and progenitor exhausted (Tpex) and terminally exhausted (Tex) subsets (right) among PD1⁺ CD8⁺ T cells in the TME. Statistical significance was determined using a two-way ANOVA followed by Tukey’s multiple comparisons test. *p < 0.05, **p < 0.01, and ***p < 0.001. The underlying numerical data for this figure can be found in S1 Data.
(TIFF)

S8 Fig Gating strategy and immune profiling of TDNL draining from Ago2 competent and deficient tumors. (**A**) Representative gating strategy for one TDLN from LLC tumor-bearing mice, collected 10 days after initiation of anti-PD1 treatment. (**B**) Flow cytometry plots showing the distribution of naive, memory, and effector CD8⁺ T cells (left), and progenitor exhausted (Tpex) and terminally exhausted (Tex) subsets (right) among PD1⁺ CD8⁺ T cells in TDLNs. Statistical significance was determined using a two-way ANOVA followed by Tukey’s multiple comparisons test. *p < 0.05, **p < 0.01, ***p < 0.001. The underlying numerical data for this figure can be found in S1 Data.
(TIFF)

S9 Fig Correlation between survival of ICI-treated NSCLC patients, Ago2 expression, and an IFN signature. (**A**) Kaplan–Meier survival curves for patients in the Stand Up To Cancer–Mark Foundation (SU2C–MARK) non-small cell lung cancer cohort stratified into high versus low Ago2 expression groups, defined using the optimal cutoff determined by maximally selected rank statistics. (**B**) Kaplan–Meier survival curves for the same patients stratified into high versus low expression groups based on a custom Ago2-dependent signature. Statistical significance was evaluated using a log-rank test (*p* < 0.05). (**C**) Optimal cutoff value for Ago2 expression in the SU2C–MARK NSCLC cohort, determined for use in Kaplan–Meier survival analysis. (**D**) Correlation between Ago2 expression levels and the custom IFN signature in patients of the same cohort. The underlying numerical data for this figure can be found in S1 Data.
(TIFF)

S1 Table Differential gene expression in wild-type versus ΔAgo2 cells and tumors. This table contains the normalized count matrix and differential expression analysis results from the bulk RNA-sequencing of wild-type and ΔAgo2 cells and tumors, indicated, respectively, as ‘in vitro’ and ‘in vivo’ in the file. The data includes the complete list of significantly upregulated and downregulated genes.
(XLSX)

S2 Table Custom IFN gene signature used for patient survival stratification. Hand-curated list of genes derived from the ex vivo bulk RNA-sequencing analysis used to define the custom IFN signature. This signature was applied to the SU2C-MARK cohort to stratify patient survival outcomes, as depicted in S9 Fig.
(XLSX)

S3 Table Differential expression of transposable elements in wild-type vs. ΔAgo2 cells. This table provides the relative expression matrix and statistical analysis of individual transposable elements (TEs). Data is derived from the same in vitro bulk RNA-sequencing dataset as S1 Table.
(XLSX)

S4 Table List of antibodies used for flow cytometry staining. (XLSX)

S1 Data Excel file containing the underlying numerical data for all figures in the main manuscript and supporting information. Each sheet corresponds to a specific figure.
(XLSX)

S1 Raw Images Original, uncropped, and minimally adjusted images supporting blot and gel results reported in the following figures: S1A and S3D Figs.
(PDF)

## Abbreviations

ACF: Acriflavine
ATA: aurintricarboxylic acid
Ago2: Argonaute 2
DMEM: Dulbecco’s Modified Eagle Medium
dsRNA: double-stranded RNA
ERVK: endogenous retrovirus K
ESC FBS: Embryonic Stem Cell fetal bovine serum
ICI: immune checkpoint inhibitors
IFN: interferons
ISGs: interferon-stimulated genes
LINEs: long interspersed nuclear elements
LLC: Lewis lung carcinoma
LTR: long terminal repeat
miRNAs: micro-RNAs
NK: natural killer
NSCLC: non-small cell lung cancer
RNAi: RNA interference
TDLNs: tumor-draining lymph nodes
TEs: transposable elements
TME: tumor microenvironment
