# Supplementary figures and images for: Argonaute 2 drives resistance to immune checkpoint inhibitors in immunorefractory non-small cell lung cancer
Source: PLoS Biol. 2026 Jun 18;24(6):e3003860. doi: 10.1371/journal.pbio.3003860 (PMC13309041; doi:10.1371/journal.pbio.3003860)

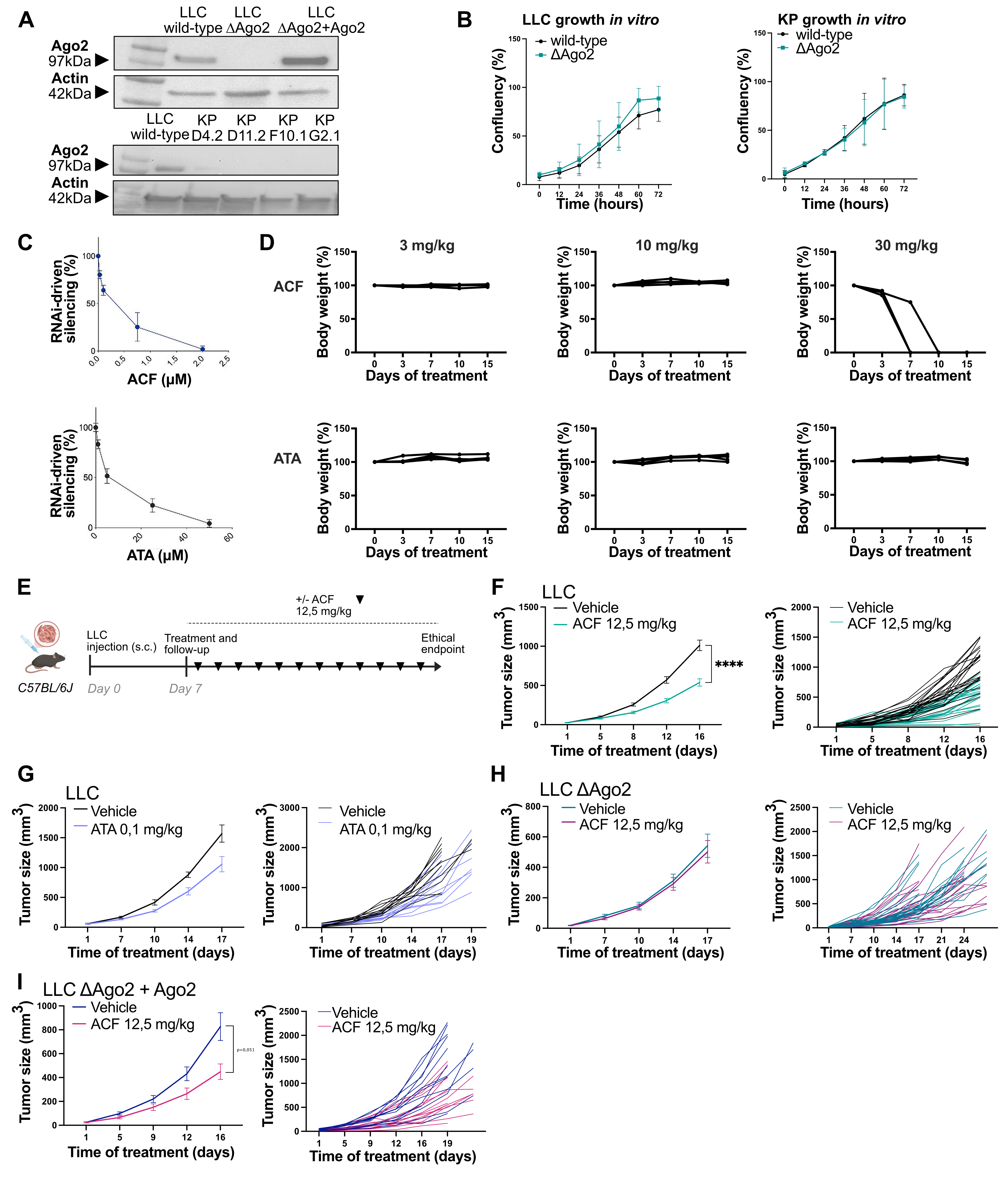

Supplement: S1 Fig — (A) Western blot quantifying Ago2 protein in LLC wild-type, LLC ΔAgo2 cells, KP ΔAgo2 cells. Clone #D11.2 was chosen for in vivo engraftment. (B) Growth rate of wild-type and ΔAgo2 LLC and KP cells, measured by percent confluency using a CellCyte live-cell imaging system (n = 3). (C) HEK293T cells expressing GFP were transfected with dsRNA targeting part of the GFP sequence, triggering RNAi-dependent GFP silencing. Cells were treated with increasing concentrations of ACF or ATA, and GFP fluorescence was quantified by flow cytometry (n = 3). (D) Body weight curves as a measure of toxicity in mice receiving ACF (3–30 mg/kg) or ATA (3–30 mg/kg), delivered intraperitoneally, 5 days per week. Four mice per group. (E) Schematics of tumor engraftment, treatment administration, and tumor growth follow-up. Created in BioRender. Poirier, E. (2026) https://BioRender.com/17igj4k (F) Wild-type mice were implanted with LLC wild-type and treated with ACF 12.5 mg/kg intraperitoneally for five days/week. Tumor growth was measured. (G) Wild-type mice were implanted with LLC wild-type and treated with ATA 0.1 mg/kg intraperitoneally for five days/week. Tumor growth was measured. (H) Wild-type mice were implanted with LLC ΔAgo2 and treated with ACF 12.5 mg/kg intraperitoneally for five days/week. Tumor growth was measured. (I) Wild-type mice were implanted with LLC ΔAgo2 + Ago2 and treated with ACF 12.5 mg/kg intraperitoneally for five days/week. Tumor growth was measured. Mean ± SEM (left) and individual tumor growth (right) are represented. (F) and (H), 24 mice per group, pooled from 3 independent experiments of 8 mice each. (G) 11 mice per group, pooled from 2 independent experiments of 5–6 mice each. (I) 14 mice per group, pooled from 2 independent experiments of 6–8 mice each. Statistical analysis was performed using two-way repeated-measures ANOVA followed by Šidák post-hoc test; *p < 0.05, **p < 0.01, and ***p < 0.001. The underlying numerical data for this figure can be found i [file pbio.3003860.s001.tiff]

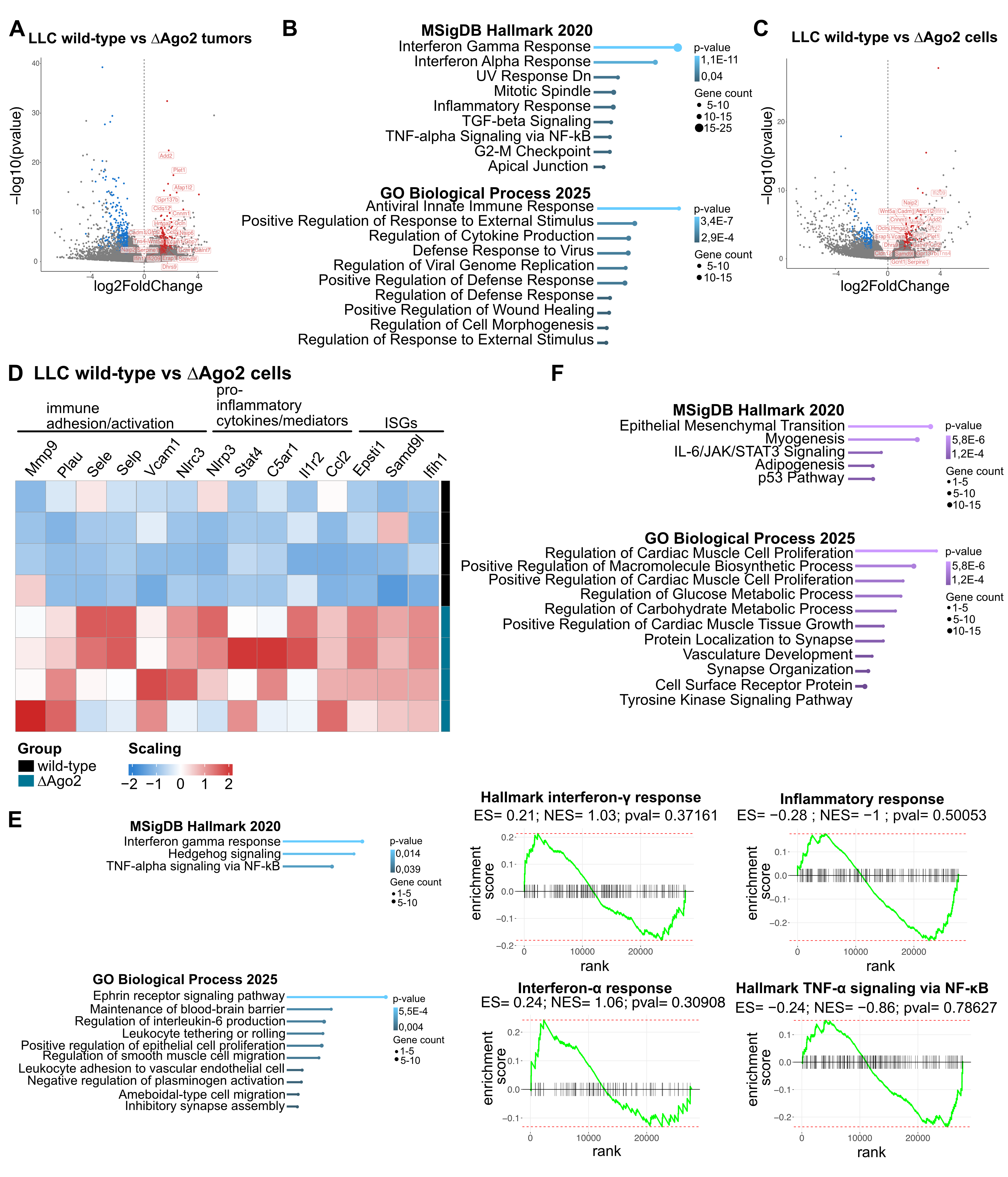

Supplement: S2 Fig — (A) Volcano plot showing significantly differentially expressed genes in tumor-derived ΔAgo2 cells compared with tumor-derived wild-type cells (p < 0.05; |log2 fold change| > 1,2). (B) Pathway enrichment analysis of significantly upregulated genes in ΔAgo2 tumor-derived cells. Data were analyzed via EnrichR. (C) Volcano plot showing significantly differentially expressed genes in ΔAgo2 versus wild-type cells cultured in vitro (p < 0.05; |log2 fold change| > 1.2). (D) Curated heatmap of selected genes upregulated in ΔAgo2 cells in vitro. (E) Pathway enrichment analysis of significantly upregulated genes in ΔAgo2 cells cultured in vitro. Data were analyzed via EnrichR and fastGSEA, with enrichment plots shown for the four most relevant pathways identified by Gene Ontology and pathway databases. (F) Pathway enrichment analysis of significantly downregulated genes in ΔAgo2 cells cultured in vitro. The underlying numerical data for this figure can be found in S1 Data. (TIFF) [file pbio.3003860.s002.tiff]

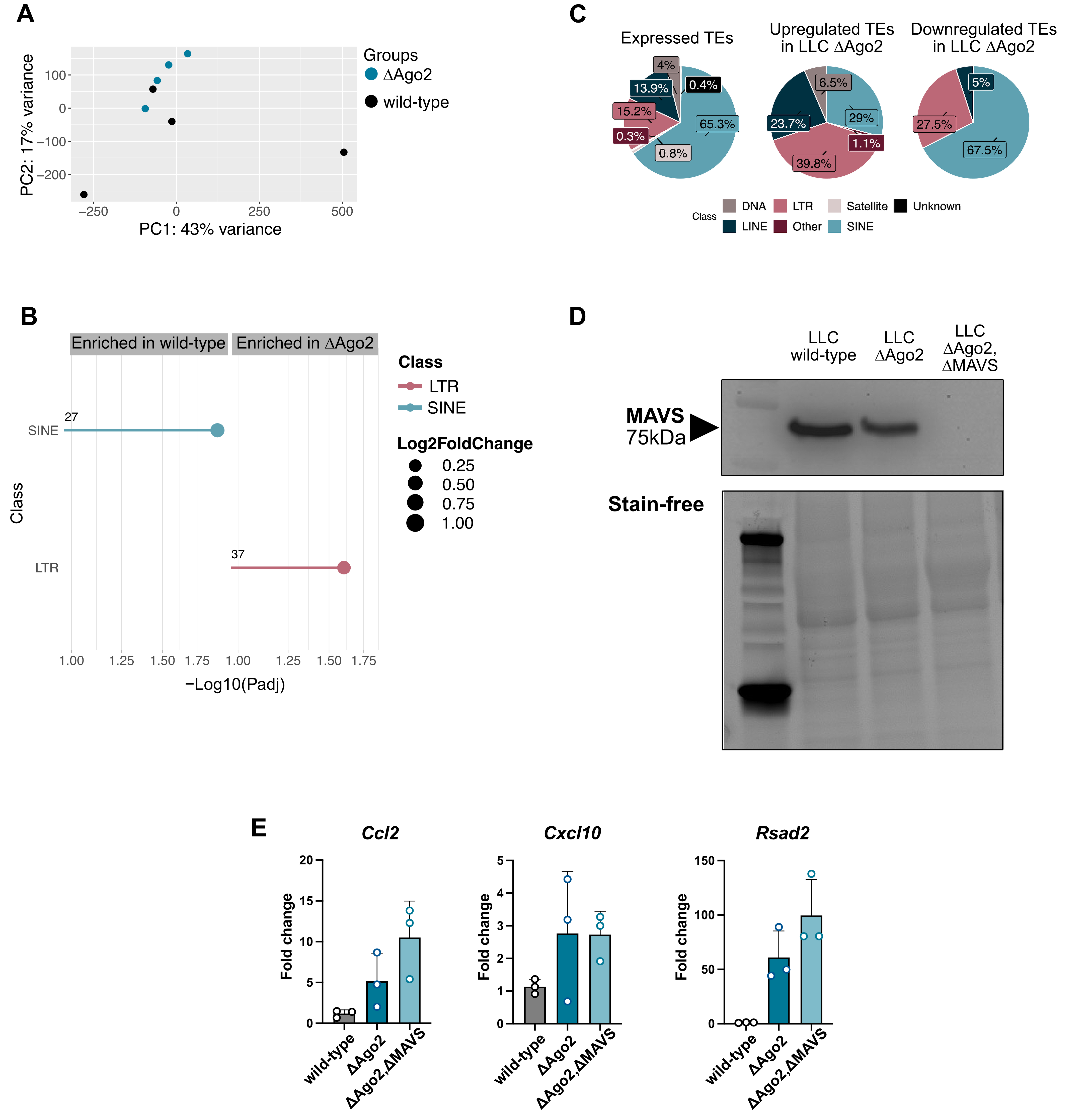

Supplement: S3 Fig — (A) Principal component analysis (PCA) of TE expression profiles in LLC wild-type and ΔAgo2 cells. (B) Relative enrichment of TE classes in wild-type and ΔAgo2 cells. (C) Distribution of expressed TE classes, showing the proportion of all TEs, upregulated TEs, and downregulated TEs in ΔAgo2 cells (left to right). (D) Western blot quantifying MAVS protein in LLC wild-type, LLC ΔAgo2 cells, LLC ΔAgo2, ΔMAVS cells. (E) Levels of Ccl2, Cxcl10, and Rsad2 transcripts measured by RT-qPCR in LLC ΔAgo2 cells and LLC ΔAgo2, ΔMAVS cells. The underlying numerical data for this figure can be found in S1 Data. (TIFF) [file pbio.3003860.s003.tiff]

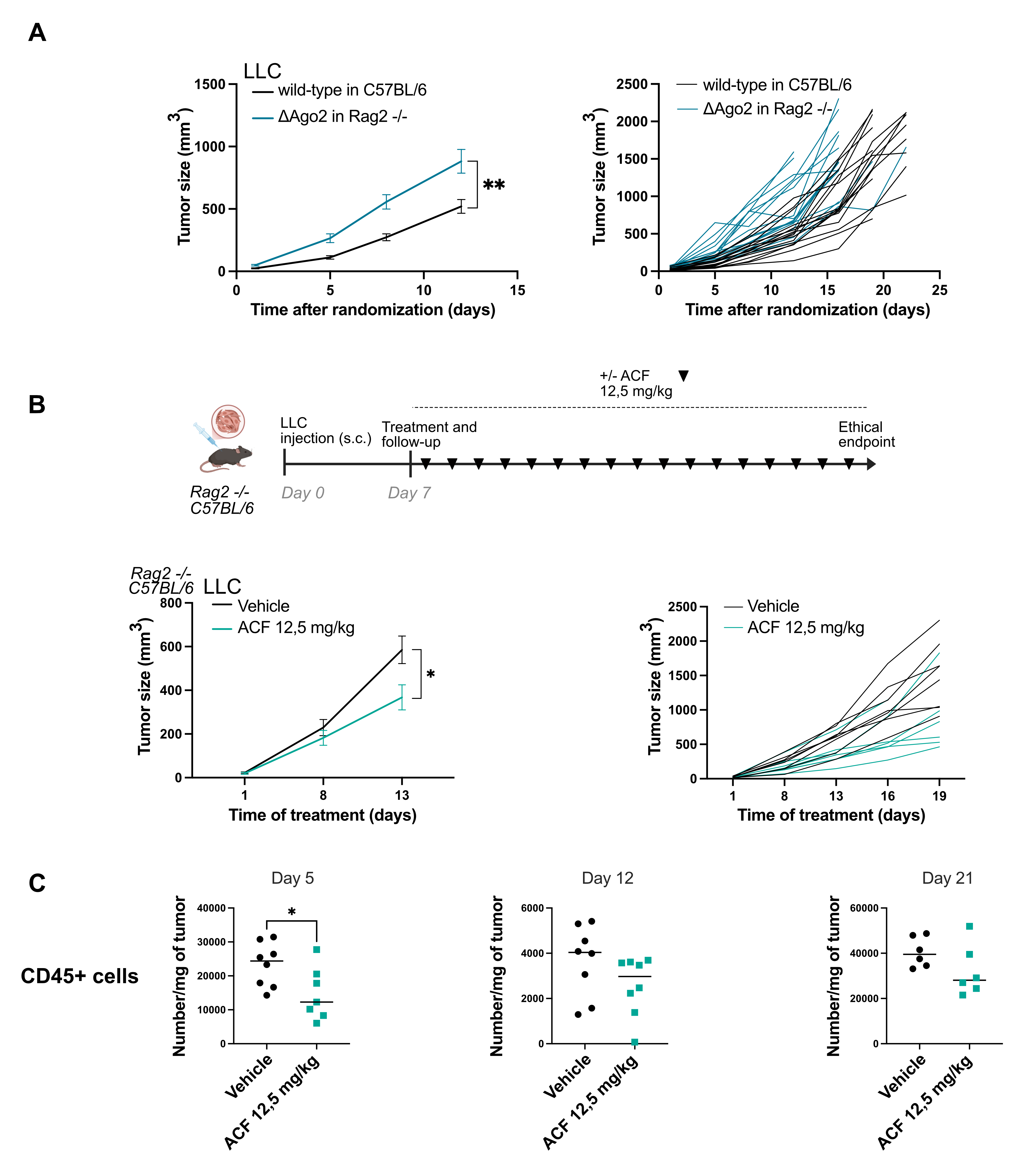

Supplement: S4 Fig — (A) Tumor growth of wild-type LLC engrafted in immunocompetent C57BL/6 mice and ΔAgo2 engrafted in Rag2−/− mice. Mean ± SEM (left) and individual tumor growth (right) are represented. (B) Schematics of tumor engraftment, treatment administration, and tumor growth follow-up. Rag2−/− mice were implanted with LLC wild-type and treated with ACF 12.5 mg/kg intraperitoneally for five days/week. Tumor growth was measured. 8 mice per group. One experiment. Created in BioRender. Poirier, E. (2026) https://BioRender.com/17igj4k. (C) Flow cytometry quantification of CD45+ immune cells in tumors treated with ACF 12.5 mg/kg intraperitoneally for five days/week and harvested at the indicated time points. Statistical analysis was performed using Mann–Whitney test; *p < 0.05, **p < 0.01, and ***p < 0.001. The underlying numerical data for this figure can be found in S1 Data. (TIFF) [file pbio.3003860.s004.tiff]

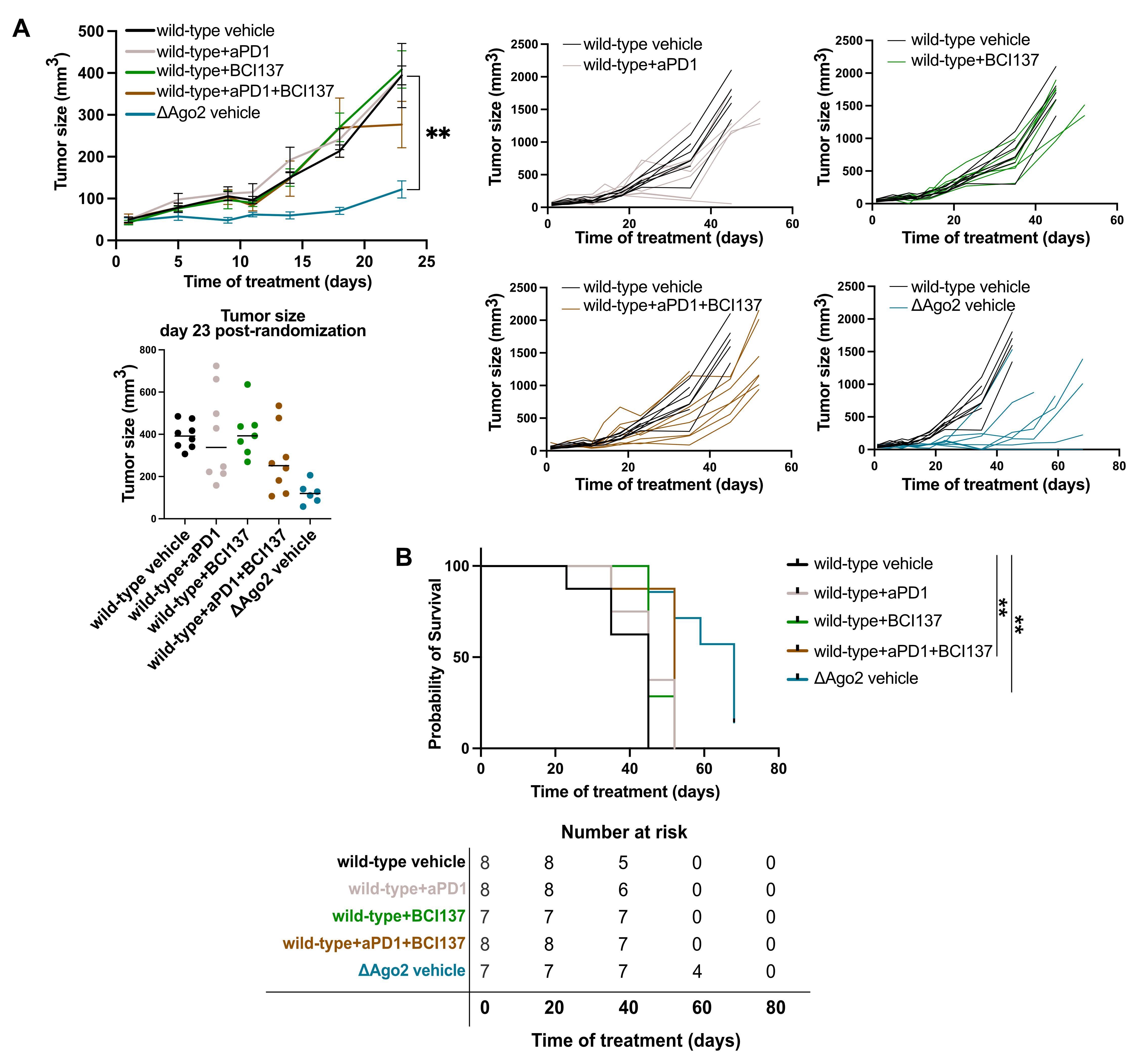

Supplement: S5 Fig — (A) Wild-type mice were implanted subcutaneously with KP wild-type and ΔAgo2 cells. Mice bearing wild-type tumors were treated with anti-PD-1 (200 µg/20 g body weight, injected intraperitoneally three times per week), BCI-137 (100 mg/kg, twice per week for the first week, then once per week for the following two weeks), or a combination of both. Mean ± SEM (left) and individual tumor growth (right) are represented. 7–8 mice per group. One experiment. Tumor sizes at day 23 post-randomization are shown. Statistical analysis was performed using Kruskall–Wallis test; *p < 0.05, **p < 0.01, and ***p < 0.001. (B) Kaplan–Meier survival curves for mice bearing wild-type tumors treated with vehicle, anti-PD1, BCI-137, or the combination, compared to mice bearing ΔAgo2 tumors. Statistical significance was evaluated using a log-rank test (p < 0.05). The underlying numerical data for this figure can be found in S1 Data. (TIFF) [file pbio.3003860.s005.tiff]

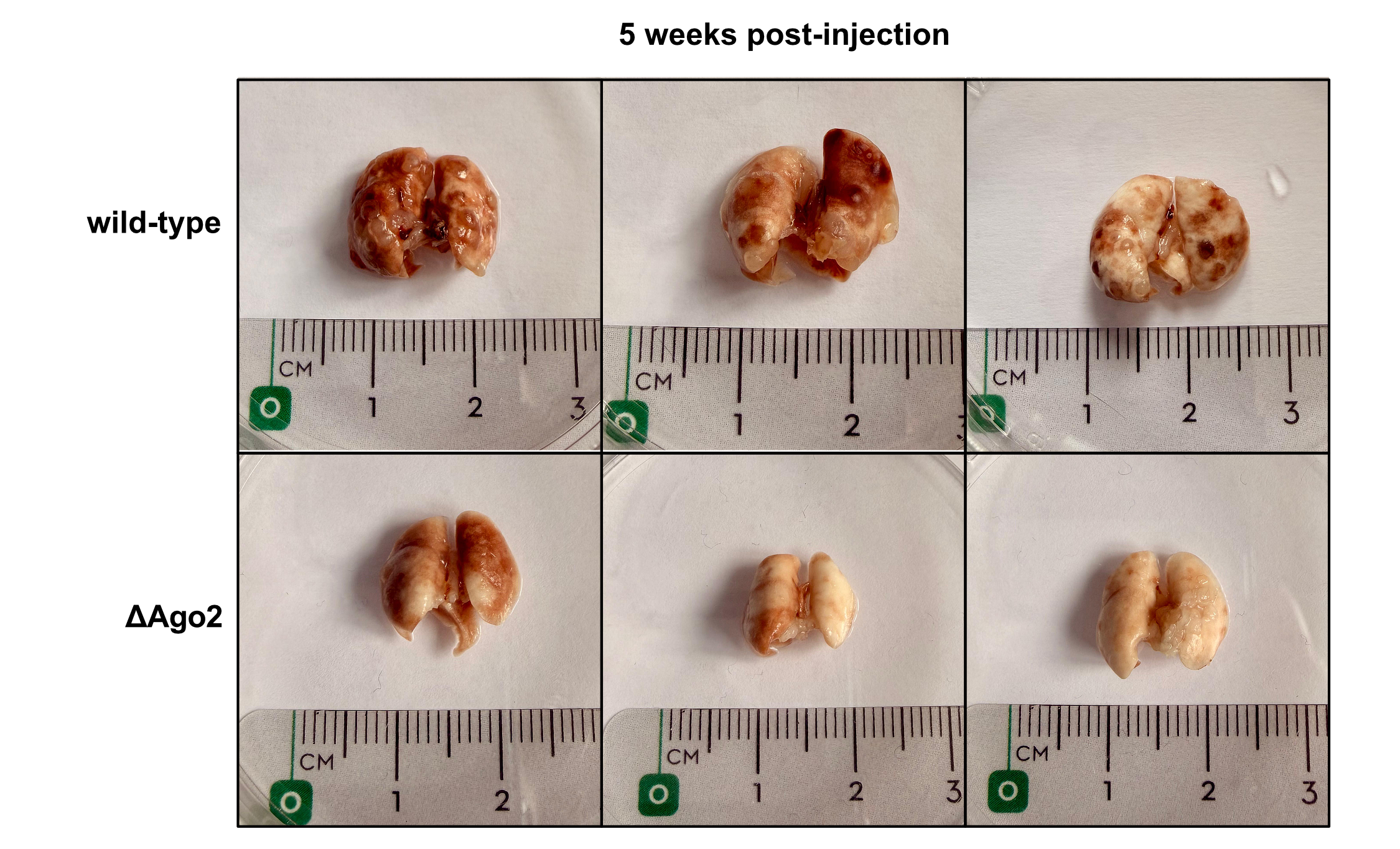

Supplement: S6 Fig — Representative macroscopic images of formalin-fixed lungs excised 5 weeks post-tail vein injection with either wild-type or ΔAgo2 KP cells. (TIFF) [file pbio.3003860.s006.tiff]

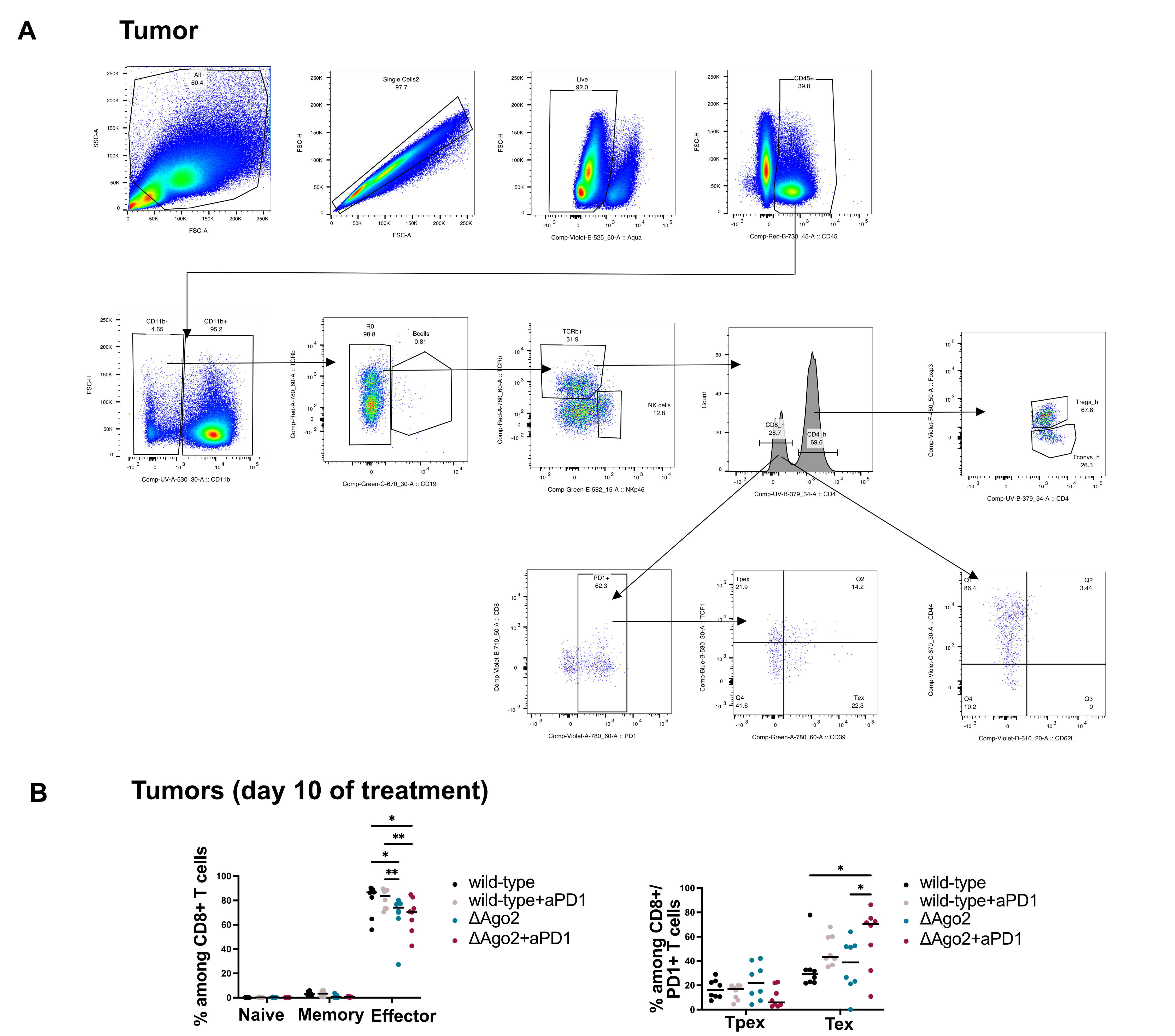

Supplement: S7 Fig — (A) Representative gating strategy for one tumor sample from LLC tumor-bearing mice, collected 10 days after initiation of anti-PD1 treatment. (B) Flow cytometry plots showing the distribution of naive, memory, and effector CD8⁺ T cells (left), and progenitor exhausted (Tpex) and terminally exhausted (Tex) subsets (right) among PD1⁺ CD8⁺ T cells in the TME. Statistical significance was determined using a two-way ANOVA followed by Tukey’s multiple comparisons test. *p < 0.05, **p < 0.01, and ***p < 0.001. The underlying numerical data for this figure can be found in S1 Data. (TIFF) [file pbio.3003860.s007.tiff]

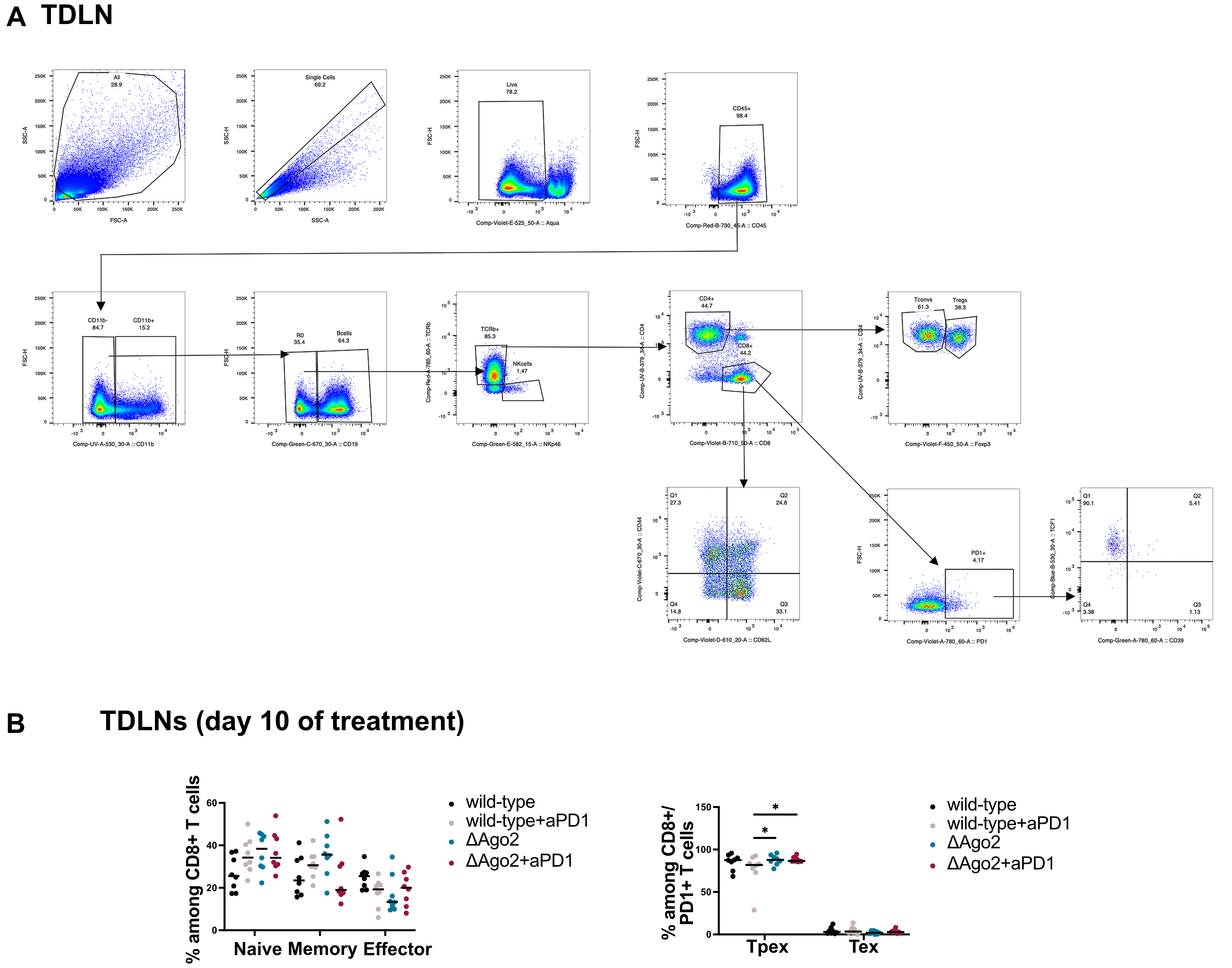

Supplement: S8 Fig — (A) Representative gating strategy for one TDLN from LLC tumor-bearing mice, collected 10 days after initiation of anti-PD1 treatment. (B) Flow cytometry plots showing the distribution of naive, memory, and effector CD8⁺ T cells (left), and progenitor exhausted (Tpex) and terminally exhausted (Tex) subsets (right) among PD1⁺ CD8⁺ T cells in TDLNs. Statistical significance was determined using a two-way ANOVA followed by Tukey’s multiple comparisons test. *p < 0.05, **p < 0.01, ***p < 0.001. The underlying numerical data for this figure can be found in S1 Data. (TIFF) [file pbio.3003860.s008.tiff]

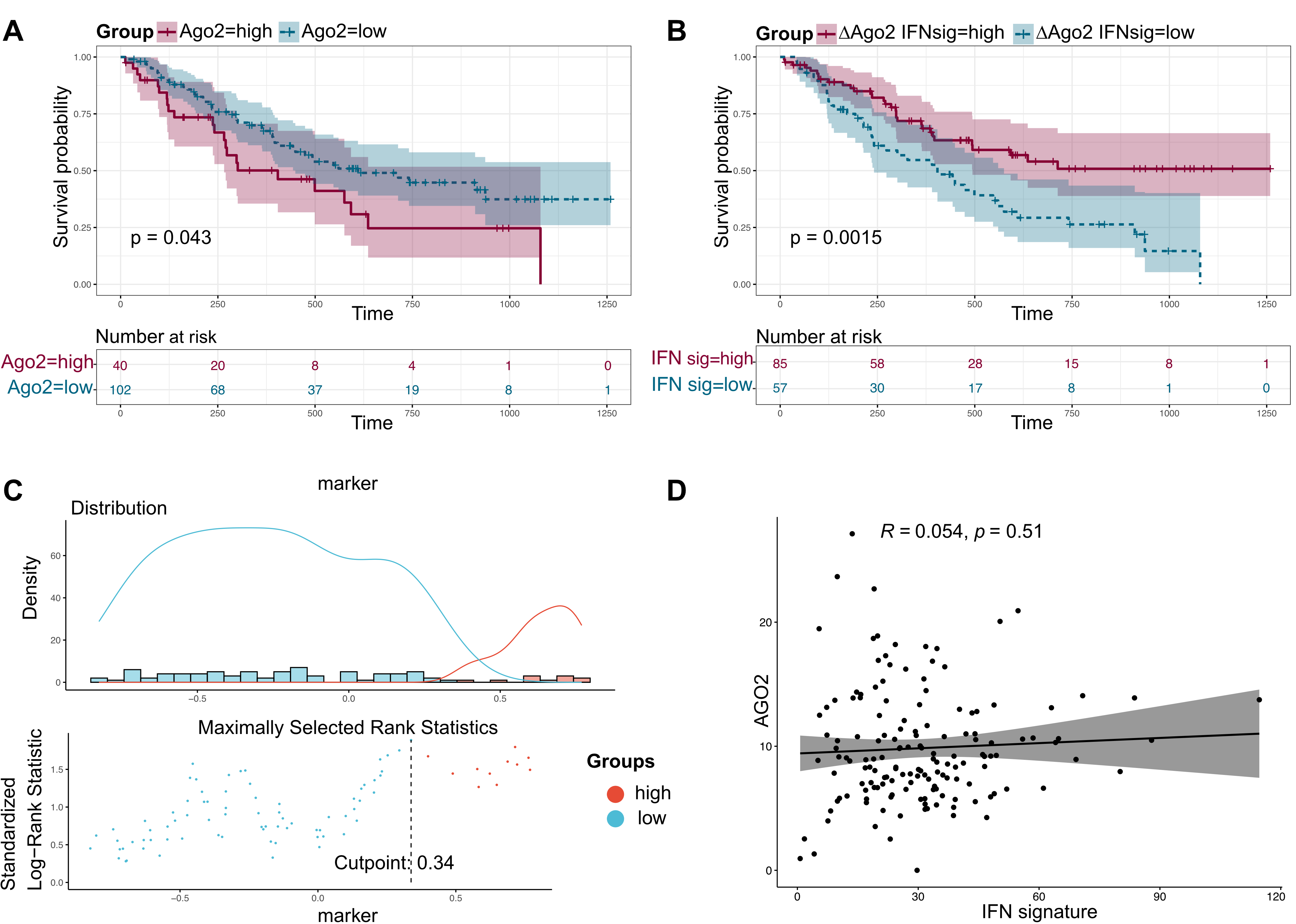

Supplement: S9 Fig — (A) Kaplan–Meier survival curves for patients in the Stand Up To Cancer–Mark Foundation (SU2C–MARK) non-small cell lung cancer cohort stratified into high versus low Ago2 expression groups, defined using the optimal cutoff determined by maximally selected rank statistics. (B) Kaplan–Meier survival curves for the same patients stratified into high versus low expression groups based on a custom Ago2-dependent signature. Statistical significance was evaluated using a log-rank test (p < 0.05). (C) Optimal cutoff value for Ago2 expression in the SU2C–MARK NSCLC cohort, determined for use in Kaplan–Meier survival analysis. (D) Correlation between Ago2 expression levels and the custom IFN signature in patients of the same cohort. The underlying numerical data for this figure can be found in S1 Data. (TIFF) [file pbio.3003860.s009.tiff]
