# Supplementary material for: Argonaute 2 drives resistance to immune checkpoint inhibitors in immunorefractory non-small cell lung cancer
Source: PLoS Biol. 2026 Jun 18;24(6):e3003860. doi: 10.1371/journal.pbio.3003860 (PMC13309041; doi:10.1371/journal.pbio.3003860)
Supplement: S1 Raw Images — S1A and S3D Figs. (PDF) [file pbio.3003860.s015.pdf]

Uncropped blot for Fig. S1A

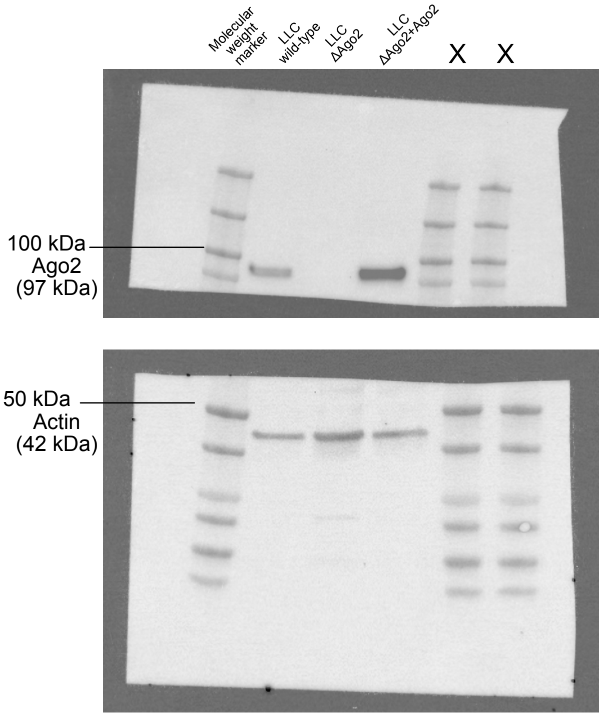

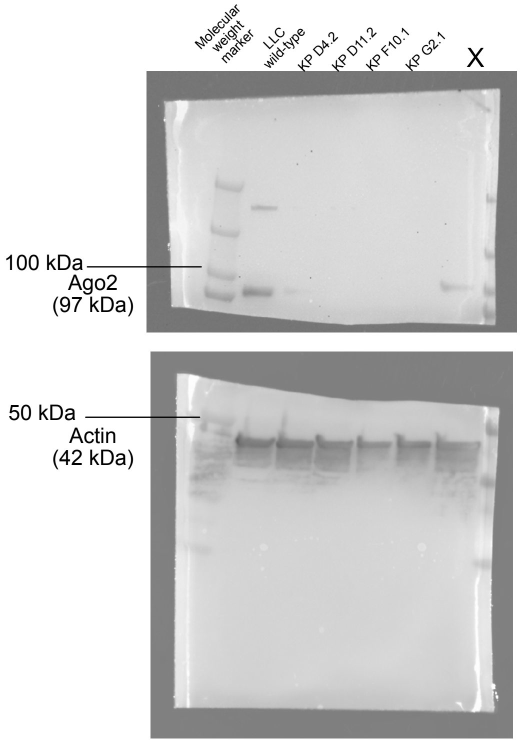

Uncropped blot for Fig. S3D

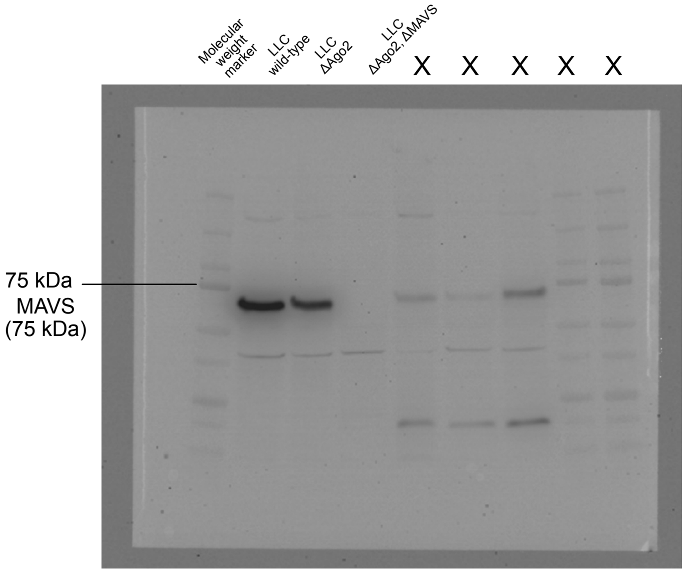

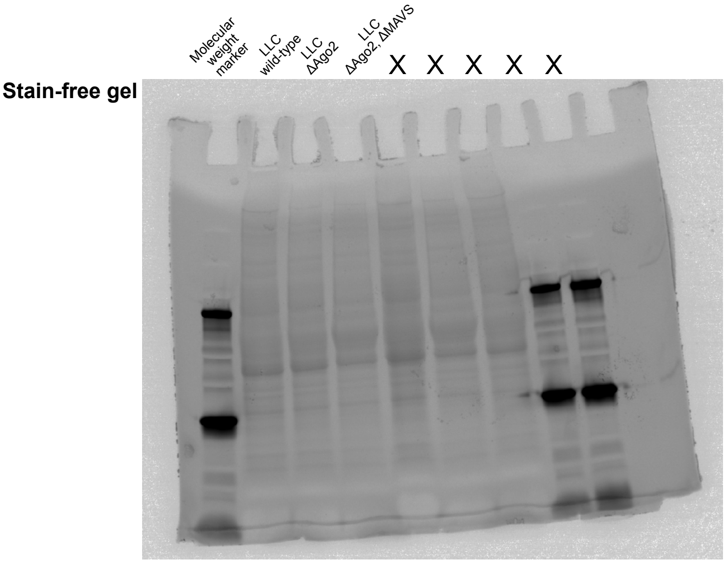

Ago2: Image acquired using a Bio-Rad ChemiDoc Go system in manual signal accumulation mode (10-second capture intervals, 3-minute total exposure).

Actin: Image acquired using a Bio-Rad ChemiDoc Go system in manual signal accumulation mode (1-second capture intervals, 60-second total exposure).

MAVS: Image acquired using a Bio-Rad ChemiDoc Go system in manual signal accumulation mode (10-second capture intervals, 3-minute total exposure).

Stain-Free Gel (Loading Control): Stain-Free gel image acquired using a Bio-Rad ChemiDoc Go system (5-minute UV activation, Stain-Free gel program).
