# Supplementary material for: Argonaute 2 drives resistance to immune checkpoint inhibitors in immunorefractory non-small cell lung cancer
Source: PLoS Biol. 2026 Jun 18;24(6):e3003860. doi: 10.1371/journal.pbio.3003860 (PMC13309041; doi:10.1371/journal.pbio.3003860)
Supplement: Attachment — Submitted filename: ResponseToReviewers_v5.pdf [file pbio.3003860.s016.pdf]

We thank the reviewers for their thoughtful and constructive comments, which have significantly improved our manuscript. **We are pleased to submit a fully revised version that addresses all reviewers' concerns.** With this revised version, we believe that we convincingly demonstrate that Ago2 represents a promising therapeutic target in cancer, particularly in combination with immune checkpoint inhibition. Our findings are complementary to those of Wang and colleagues and place Ago2 at the center of tumor–immune interactions across multiple cancer contexts. At the same time, our results indicate that currently available small-molecule Ago2 inhibitors remain suboptimal for *in vivo* use, underscoring the need for the development of more specific and potent compounds. Although the identification and optimization of such inhibitors lie beyond the scope of the present study, our work establishes a strong foundation for the development of Ago2-targeted immunotherapeutic strategies.

The major additions and revisions include the following highlights:

1) Mechanistic insight into Ago2-dependent inflammation. We provide data supporting the mechanism by which loss of Ago2 drives inflammation in cancer cells. This includes direct demonstration of the immunogenicity of dsRNA accumulating in Ago2-deficient cells, as well as evidence implicating RIG-I/MDA5 and TLR3 as the pattern recognition receptors responsible for sensing this dsRNA.

2) Orthotopic lung cancer model. We introduce an orthotopic model of lung cancer in which loss of Ago2 results in a striking reduction in tumor burden, thereby extending our findings to tumors growing within their native tissue context. These results further underscore the therapeutic potential of targeting Ago2 for antitumor intervention.

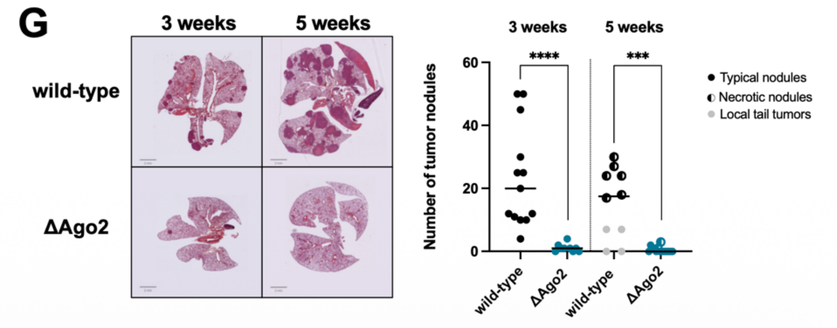

Fig 3G. Representative hematoxylin and eosin staining of lung sections from wild-type mice at 3 and 5 weeks post-injection with either wild-type or ΔAgo2 KP cells. Microscopic quantification of tumor nodules was performed by a blinded expert pathologist.

3) Pharmacological inhibition of Ago2. We evaluated the Ago2 inhibitor BCI-137, previously described by Wang et al. (DOI: 10.1016/j.celrep.2025.115445), in combination with immune checkpoint blockade. In their study, Wang and colleagues reported a synergistic interaction between BCI-137 and ICI in immunologically 'hot' syngeneic tumor models that are responsive to checkpoint inhibition. In our model, we likewise observed synergy between the two treatments, although the magnitude of the effect was more modest, as expected for 'cold' tumors.

Reviewer #1

This study provides a compelling investigation into the role of Ago2 in mediating resistance to immune checkpoint inhibitors (ICI) in non-small cell lung cancer (NSCLC), linking it mechanistically to interferon signaling suppression and reduced tumor immunogenicity. The findings are potentially significant and may provide new therapeutic avenues for converting immunologically 'cold' NSCLC tumors into 'hot' ones. However, several major concerns regarding the study's novelty, mechanistic depth, and conceptual clarity need to be adequately addressed before the manuscript can be considered for publication.

1. The authors state that "Ago2 is a driver of tumor growth" (line 145). If so, would overexpression of Ago2 be sufficient to promote tumor progression?

This concern is addressed by our rescue experiments, in which Ago2-deficient cells were complemented with exogenous Ago2 expression (Fig 1B-C and S1A). Although rescued cells express higher levels of Ago2 than parental LLC cells (Fig S1A), they nonetheless form tumors with growth kinetics indistinguishable from wild-type controls (Fig 1B). We interpret these findings to indicate that Ago2 functions enzymatically, such that endogenous Ago2 levels in wild-type cells are sufficient for maximal activity, and increased expression does not confer a gain-of-function phenotype.

2. Given that Ago2 is a key player in the RNAi pathway, does its knockout (as shown in Figure 2) result in global dysregulation of gene expression across the genome—beyond the interferon signaling pathway?

Thank you for this suggestion. In addition to genes with upregulated expression, we have now updated the analysis to include genes whose expression is downregulated in Ago2-deficient cells relative to wild-type cells, as identified by RNA-seq (Fig S2F). Pathway analysis reveals reduction in genes linked to epithelial-mesenchymal transition, as well as genes involved in metabolic processes (e.g., glucose metabolic processes, myo- and adipogenesis) and impact on signaling pathways such as IL-6/JAK/STAT3 and p53 axes.

3. Is the role of Ago2 in immunotherapy resistance solely through immune suppression, or does it have a dual function that also involves promoting tumor cell proliferation? Specifically, did the authors compare the proliferation rates of Ago2-KO and wild-type (WT) cells in vitro?

We have now included these data to address this point, demonstrating that cells lacking Ago2 grow at levels comparable to wild-type cells (Fig S1B). We further show that Ago2-deficient KP cells grow to wild-type levels in *Rag2*$^{-/-}$ mice, excluding a cell-intrinsic role for Ago2 in tumor cell proliferation in this context (Fig 3C).

In Figure 3B, using Rag2−/−mice, Ago2 knockout and WT tumors exhibited comparable growth rates, suggesting that Ago2 loss may not significantly affect tumor growth in NSCLC. However, in hepatocellular carcinoma, Ago2 upregulation has been shown to enhance tumor cell proliferation, migration, and metastatic potential in vivo. How can these tissue-specific differences in Ago2 function be reconciled?

There are indeed notable differences between NSCLC and HCC, which may in part reflect the distinct tissue origins of these cancers. miRNA expression patterns have been shown to be highly tissue-specific (DOI: 10.1093/nar/gkw116), resulting in divergent miRNA-driven regulatory networks across organs. Consequently, perturbation of miRNA-mediated gene regulation in cells of hepatic origin may impact cellular proliferation and homeostasis differently than in lung-derived cells.

Furthermore, since treatment with ACF and ATA inhibitors suppresses tumor growth in Ago2-dependent models, is this effect solely due to off-target actions, or might Ago2 also directly influence tumor cell proliferation?

We now provide additional data showing that loss of Ago2 does not affect the proliferation of LLC or KP cells in vitro (Fig S1B). Consistently, Ago2-deficient KP cells grow to wild-type levels in Rag2 KO mice (Fig 3C). These results argue against a role for Ago2 in regulating cell proliferation in this context. Instead, we propose that the phenotypes observed with ACF treatment may be partially explained by its documented off-target effects on DNA topoisomerases, key components of the DNA replication machinery required for cell division, which could impair proliferation independently of Ago2 inhibition (DOI: 10.1016/j.omtn.2022.01.016).

4. Wang et al. have recently reported that Ago2 mediates immunosuppression by inhibiting IFN-γ signaling, thereby contributing to immunotherapy failure. What novel mechanistic or conceptual insight does the present study provide regarding Ago2-mediated immune suppression?

Our study demonstrates that the role of Ago2 in restraining antitumor immune responses also operates in immunologically ‘cold’ tumors, which typically respond poorly to immune checkpoint inhibition (ICI). Accordingly, our data suggest that Ago2 represents a potential therapeutic target for enhancing the efficacy of ICI in patients who are refractory to treatment. In addition, we uncover a distinct molecular mechanism compared with that proposed by Wang and colleagues: Ago2 limits the expression of transposable elements. In its absence, TE-derived dsRNA accumulates, triggering an inflammatory response that enhances antitumor immunity.

5. The mechanism underlying Ago2-mediated immune suppression remains incompletely elucidated. The authors propose that Ago2 inhibits cytoplasmic dsRNA accumulation, thereby suppressing IFN signaling; however, what is the precise molecular mechanism involved?

Thank you for this question. We have strengthened the molecular mechanism presented in Fig 2 by providing additional data. We now demonstrate that dsRNA accumulating in the absence of Ago2 is immunostimulatory when introduced into wild-type cells. Furthermore, we provide evidence that this dsRNA is likely sensed by the pattern recognition receptors RIG-I/MDA5 and TLR3.

Additionally, while the model suggests that Ago2 suppresses TE transcription to inhibit ERVK and innate immune activation, Figures 4 and 5 focus on adaptive immunity. The direct relevance of these findings to the proposed innate immune pathway is not clearly established.

We apologize for the lack of clarity. We have now explicitly underlined the link between IFN-driven cytokine production by tumor cells and the enhancement of adaptive immune responses (lines 240–244) : « Altogether, these data demonstrate that Ago2 loss induces a tumor-intrinsic

inflammatory state, including elevated expression of the chemokines CCL2 and CXCL10, which are known to promote myeloid and T cell recruitment in tumors [11,14]. This Ago2-dependent pro-inflammatory state may therefore have an impact on the antitumoral immune response »

Reviewer #2

The manuscript titled "Ago2 loss restores sensitivity to immune checkpoint inhibitors in immunorefractory lung cancer" by Anobile et al. presents an interesting study investigating the role of Argonaute 2 (Ago2) in driving resistance to immune checkpoint inhibitor (ICI) therapy in non-small cell lung cancer (NSCLC). The authors utilize two well-established immunologically "cold" and ICI-resistant murine NSCLC models (LLC and KP). They demonstrate that genetic deletion of Ago2 in tumor cells significantly impairs tumor growth in immunocompetent mice but not in Rag2-deficient mice, highlighting the essential role of the adaptive immune system in this effect. Crucially, Ago2 loss sensitizes these otherwise refractory tumors to anti-PD-1 therapy, leading to markedly reduced tumor growth and even complete regressions in the KP model.

Mechanistically, the authors provide evidence that Ago2 deficiency leads to an upregulation of an interferon (IFN) signature and pro-inflammatory cytokines in tumors, both in vivo and in cell culture. They link this phenotype to the derepression of transposable elements (TEs) and a consequent accumulation of immunostimulatory double-stranded RNA (dsRNA) in Ago2-deficient cells. Flow cytometry analyses reveal that Ago2 loss, especially when combined with anti-PD-1, reshapes the tumor microenvironment, promoting the infiltration of immune cells, including CD8+ T cells, and fostering a more favorable T cell differentiation state. Finally, the clinical relevance is supported by analyzing an NSCLC patient cohort, showing that high tumor Ago2 expression correlates with poor survival after ICI treatment, while a high "Ago2-dependent" IFN gene signature correlates with improved survival.

Overall, this paper is well written, yet the relevance of the proposed mechanism is not sufficiently clear at this stage. There are a few major issues identified which are listed below that the authors should consider addressing.

1. In Figure1, authors adopted Ago2-knocked out cell lines. However, the current model does not rule out the possibility that Ago2 loss intrinsically impairs tumor cell growth, independent of the immune system. The use of Rag2⁻/⁻ mice, while informative, is not sufficient to dissect this potential confounder. To conclusively demonstrate that the restored ICI sensitivity is due to a reversible, immune-mediated mechanism, the authors should employ an inducible knockdown system (e.g., Doxycycline-inducible shRNA) to deplete Ago2 after tumors have been established.

To thoroughly address the reviewer's point, we now provide analyses of wild-type and ΔAgo2 LLC and KP cells *in vitro*, demonstrating that loss of Ago2 does not impair cell growth (Fig S1B). We additionally assessed the growth of KP cells either competent or deficient for Ago2 in $Rag2^{-/-}$ mice. Consistent with our findings in the LLC model, wild-type and Ago2-deficient KP tumors grew at comparable rates in this immune-deficient context (Fig 3C). We were unable to perform the doxycycline-inducible shRNA experiments within the six-month revision period. Nevertheless, we believe that the revised dataset, which includes both *in vitro* and *in vivo* analyses across two independent NSCLC models, provides strong functional evidence that loss of Ago2 does not affect tumor cell proliferation. Instead, these results implicate adaptive immunity as the primary determinant of the growth defect observed *in vivo*.

2. The study lacks investigation into the therapeutic potential of combining an Ago2 inhibitor with anti-PD-1 antibody. To address this, experiments combining an Ago2 inhibitor with anti-PD-1 therapy should be performed in both the LLC and KP animal models to demonstrate synergistic efficacy.

Thank you for this suggestion. To address this point, we evaluated the therapeutic efficacy of combining an Ago2 inhibitor with anti-PD-1 treatment in the KP model, which showed the most pronounced effect of Ago2 genetic deletion on tumor growth. We used the Ago2 inhibitor BCI-137, previously reported by Wang and colleagues to synergize with immune checkpoint inhibition in immunologically 'hot' tumor models, and followed a comparable dosing regimen.

In our immunologically 'cold' KP tumor model, neither BCI-137 nor anti-PD-1 monotherapy were effective; however the combination of BCI-137 and anti-PD-1 resulted in a modest reduction in tumor burden and an extension of overall survival (Fig S4), indicating that Ago2 inhibition can sensitize otherwise checkpoint-refractory tumors to immune therapy.

The magnitude of the therapeutic benefit observed here is more limited than that reported in prior studies using immunologically 'hot' tumor models. This difference may reflect tissue-dependent activity of BCI-137, tumor-type–specific requirements for Ago2 inhibition, or the distinct tumor microenvironment of 'cold' lung tumors compared to more inflamed models.

Nevertheless, we would argue that the development of a potent and selective small-molecule inhibitor of Ago2 represents a long-term effort that lies beyond the scope of the present study. Instead, our work provides a comprehensive genetic, molecular, and functional framework establishing Ago2 as a therapeutically actionable target in cancer, particularly when combined with immune checkpoint inhibition. We anticipate that, together with the work of Wang and colleagues, these findings will open new avenues for the development of next-generation Ago2 inhibitors suitable for therapeutic application.

3. In Figure4, authors demonstrated that knocking-out Ago2 in tumor cells influenced the immune infiltration in TDLNs. These findings are not fully compelling. To strengthen the study, the authors must address several key points:

1) Verification of TDLNs: They must provide experimental evidence, such as a dye injection assay, to conclusively demonstrate that the analyzed lymph nodes are indeed the tumor-draining lymph nodes (TDLNs).

To experimentally verify tumor drainage, we performed a tumor-proximal Evans Blue (5%) injection assay and identified the lymph nodes that directly received lymphatic drainage from the tumor, confirming that the analyzed lymph nodes in our experimental procedures correspond to bona fide tumor-draining lymph nodes.

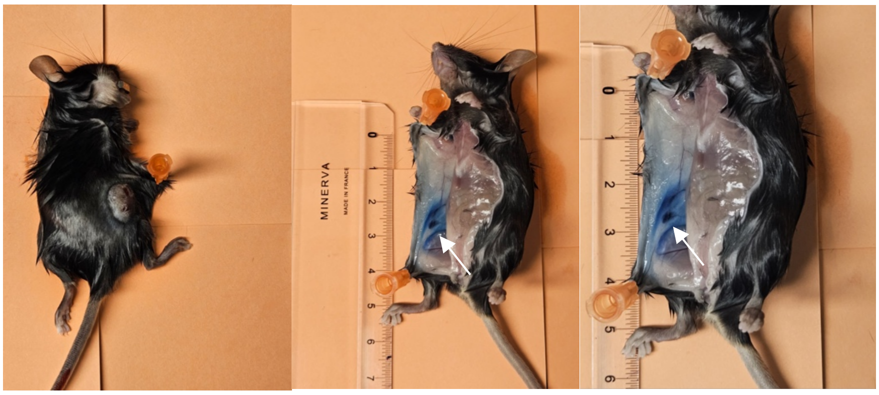

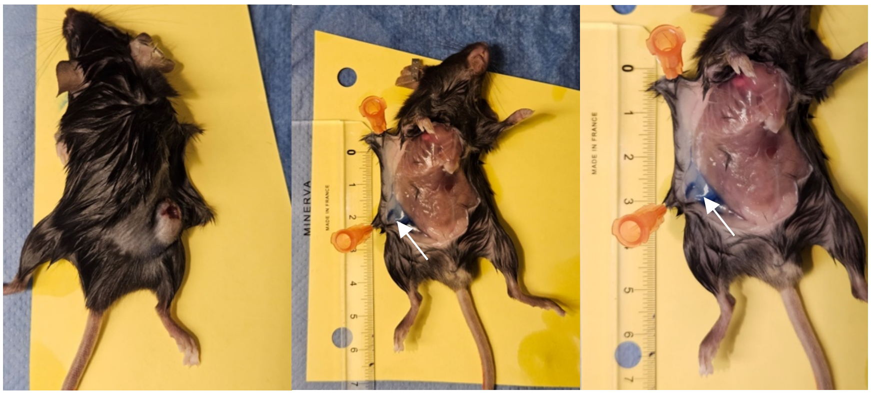

White arrow: inguinal lymph node, draining the ipsilateral subcutaneous KP tumor. Mice used: immunocompetent C57BL/6J.

2) Orthotopic Model Validation: The observations need to be validated in an orthotopic lung cancer model, as the tumor microenvironment and lymphatic drainage in a subcutaneous model may not faithful.

Thank you for this suggestion. We established an orthotopic lung cancer model in which KP cells are injected via the tail vein, allowing them to home and colonize the lung. Tumor burden was assessed at 3 and 5 weeks post-injection by hematoxylin and eosin staining of lung sections. Whereas wild-type KP cells generated an average of 15 to 20 tumors per animal, KP cells lacking Ago2 failed to produce detectable tumors in the majority of mice (Fig 3G). Consistent with our findings in syngeneic models, these findings demonstrate that Ago2 promotes lung cancer growth within its native tissue context.

3) Molecular Mechanism: A more detailed molecular mechanism is needed to connect Ago2 loss to the observed immune activation. The proposed link involving transposable elements and dsRNA requires functional validation, as suggested in previous comments.

We have strengthened the molecular mechanism presented in Fig 2 by providing additional data. We now demonstrate that dsRNA accumulating in the absence of Ago2 is immunostimulatory when introduced into wild-type cells. Furthermore, we provide evidence that this dsRNA is likely sensed by the pattern recognition receptors RIG-I/MDA5 and TLR3.

Reviewer #3

The present manuscript, titled "Ago2 loss restores sensitivity to immune checkpoint inhibitors in immunorefractory lung cancer," presents a significant finding. Genetic Ago2 deletion was observed to reduce tumor growth in immunocompetent animals. Notably, Ago2 deletion restored defective Type I interferon signaling and sensitized tumors to PD-1 immune checkpoint blockade.

The manuscript is logically structured, but it could benefit from linguistic improvements to enhance its readability.

Thank you for this suggestion. We have revised several parts of the manuscript to improve clarity and readability.

Overall, the presented results are of great scientific interest and have already been validated by others, further substantiating the findings. However, the translational value of the results is somewhat limited due to the off-target cytotoxicity exhibited by acriflavine in immunodeficient mice. It would have been beneficial to explore the use of aurintricarboxylic acid, which demonstrated less cytotoxic effects while simultaneously exhibiting tumor growth-inhibiting effects, even at low concentrations. Chemical inhibition of Ago2 (by potentially ATA, or BCI-137 as Wang et al. did) combined with PD-1 immune checkpoint blockade holds substantial translational promise.

Thank you for this suggestion. To address this point, we evaluated the therapeutic efficacy of combining an Ago2 inhibitor with anti-PD-1 treatment in the KP model, which showed the most pronounced effect of Ago2 genetic deletion on tumor growth. We used the Ago2 inhibitor BCI-137, previously reported by Wang and colleagues to synergize with immune checkpoint inhibition in immunologically ‘hot’ tumor models, and followed a comparable dosing regimen.

In our immunologically ‘cold’ KP tumor model, neither BCI-137 nor anti-PD-1 monotherapy were effective; however the combination of BCI-137 and anti-PD-1 resulted in a modest reduction in tumor burden and an extension of overall survival (Fig S4), indicating that Ago2 inhibition can sensitize otherwise checkpoint-refractory tumors to immune therapy.

The magnitude of the therapeutic benefit observed here is more limited than that reported in prior studies using immunologically ‘hot’ tumor models. This difference may reflect tissue-dependent activity of BCI-137, tumor-type–specific requirements for Ago2 inhibition, or the distinct tumor microenvironment of ‘cold’ lung tumors compared to more inflamed models.

Nevertheless, we would argue that the development of a potent and selective small-molecule inhibitor of Ago2 represents a long-term effort that lies beyond the scope of the present study. Instead, our work provides a comprehensive genetic, molecular, and functional framework establishing Ago2 as a therapeutically actionable target in cancer, particularly when combined with immune checkpoint inhibition. We anticipate that, together with the work of Wang and colleagues, these findings will open new avenues for the development of next-generation Ago2 inhibitors suitable for therapeutic application.
