# Supplementary material for: Argonaute 2 drives resistance to immune checkpoint inhibitors in immunorefractory non-small cell lung cancer
Source: PLoS Biol. 2026 Jun 18;24(6):e3003860. doi: 10.1371/journal.pbio.3003860 (PMC13309041; doi:10.1371/journal.pbio.3003860)
Supplement: Peer review history

# Editor report: Decision Letter 0

**DOI:** 10.1371/journal.pbio.3003860.r001

**Authors:** Richard Hodge

12 Sep 2025

Dear Dr Poirier,

Thank you for submitting your manuscript entitled "Ago2 loss restores sensitivity to immune checkpoint inhibitors in immunorefractory lung cancer" for consideration as a Research Article by PLOS Biology.

Your manuscript has now been evaluated by the PLOS Biology editorial staff, as well as by an academic editor with relevant expertise, and I am writing to let you know that we would like to send your submission out for external peer review under our complementary research policy.

IMPORTANT: After discussions with the rest of the team, we would like to consider your manuscript as a 'Short Report' at the journal. Upon resubmission (guidelines below), I would be grateful if you could please tick 'Short Report' as the article type in the dropdown menu in the online submission form.

Before we can send your manuscript to reviewers, we need you to complete your submission by providing the metadata that is required for full assessment. To this end, please login to Editorial Manager where you will find the paper in the 'Submissions Needing Revisions' folder on your homepage. Please click 'Revise Submission' from the Action Links and complete all additional questions in the submission questionnaire.

Once your full submission is complete, your paper will undergo a series of checks in preparation for peer review. After your manuscript has passed the checks it will be sent out for review. To provide the metadata for your submission, please Login to Editorial Manager (https://www.editorialmanager.com/pbiology) within two working days, i.e. by Sep 14 2025 11:59PM.

If your manuscript has been previously peer-reviewed at another journal, PLOS Biology is willing to work with those reviews in order to avoid re-starting the process. Submission of the previous reviews is entirely optional and our ability to use them effectively will depend on the willingness of the previous journal to confirm the content of the reports and share the reviewer identities. Please note that we reserve the right to invite additional reviewers if we consider that additional/independent reviewers are needed, although we aim to avoid this as far as possible. In our experience, working with previous reviews does save time.

If you would like us to consider previous reviewer reports, please edit your cover letter to let us know and include the name of the journal where the work was previously considered and the manuscript ID it was given. In addition, please upload a response to the reviews as a 'Prior Peer Review' file type, which should include the reports in full and a point-by-point reply detailing how you have or plan to address the reviewers' concerns.

During the process of completing your manuscript submission, you will be invited to opt-in to posting your pre-review manuscript as a bioRxiv preprint. Visit http://journals.plos.org/plosbiology/s/preprints for full details. If you consent to posting your current manuscript as a preprint, please upload a single Preprint PDF.

Feel free to email us at plosbiology@plos.org if you have any queries relating to your submission.

Kind regards,

Richard

Richard Hodge, PhD

Senior Editor, PLOS Biology

rhodge@plos.org

PLOS

Empowering researchers to transform science

Carlyle House, Carlyle Road, Cambridge, CB4 3DN, United Kingdom

California (U.S.) corporation #C2354500, based in San Francisco

# Peer review: Decision Letter 1

**DOI:** 10.1371/journal.pbio.3003860.r002

**Authors:** Richard Hodge

30 Oct 2025

Dear Enzo,

Thank you for your patience while your manuscript "Ago2 loss restores sensitivity to immune checkpoint inhibitors in immunorefractory lung cancer" was peer-reviewed at PLOS Biology as a Short Report. Please accept my sincere apologies for the delays that you have experienced during the peer review process. Your manuscript has been evaluated by the PLOS Biology editors, an Academic Editor with relevant expertise, and by three independent reviewers.

As you will see in the reviewer reports, which can be found at the end of this email, although the reviewers find the work potentially interesting, they have also raised a substantial number of important concerns. Based on their specific comments and following discussion with the Academic Editor, it is clear that a substantial amount of work would be required to meet the criteria for publication in PLOS Biology. However, given our and the reviewer interest in your study, we would be open to inviting a comprehensive revision of the study that addresses the reviewers' comments. Given the extent of revision that would be needed, we cannot make a decision about publication until we have seen the revised manuscript and your response to the reviewers' comments. Your revised manuscript would need to be seen by the reviewers again, but please note that we would not engage them unless their main concerns have been addressed.

As you can see, the reviewers are generally interested in the findings, but they raise some important and overlapping concerns. Reviewer’s #1 and #2 raise concerns about the overall strength of the mechanistic insights connecting Ago2 loss to immune activation and Reviewer #2 notes that the proposed link involving transposable elements and dsRNA requires further functional validation. In addition, the reviewers note that the data does not rule out the possibility that Ago2 loss impairs tumor growth and they ask that experiments using an inducible knockdown system to deplete Ago2 after tumors have been established are included. Finally, Reviewer’s #2 and #3 both raise concerns about the therapeutic relevance of the findings and ask that experiments combining an Ago2 inhibitor with anti- PD-1 are performed.

After discussing the reviews with the academic editor, given the Short Report format, we do not necessarily require a fully fleshed out mechanism for Ago2-mediated immune suppression. However, we think it would be beneficial to include some preliminary insights in the revised version in line with the reviewer comments. We would also consider testing the therapeutic relevance of the findings with an anti Ago2/PD-1 combination therapy essential for publication in the journal. Although several reviewers raise concerns with the conceptual novelty of the study given previous work by Wang et al, please note that any novelty concerns have bot factored into our editorial evaluation since your study is being considered under our scooping protection policy.

In addition to these revisions, you will need to complete some formatting changes, which you will receive in a follow up email. A member of our team will be in touch with a set of requests shortly.

We appreciate that these requests represent a great deal of extra work, and we are willing to relax our standard revision time to allow you 6 months to revise your study. Please email us (plosbiology@plos.org) if you have any questions or concerns, or envision needing a (short) extension.

At this stage, your manuscript remains formally under active consideration at our journal; please notify us by email if you do not intend to submit a revision so that we may withdraw it.

**IMPORTANT - SUBMITTING YOUR REVISION**

Your revisions should address the specific points made by each reviewer. Please submit the following files along with your revised manuscript:

1. A 'Response to Reviewers' file - this should detail your responses to the editorial requests, present a point-by-point response to all of the reviewers' comments, and indicate the changes made to the manuscript.

*NOTE: In your point by point response to the reviewers, please provide the full context of each review. Do not selectively quote paragraphs or sentences to reply to. The entire set of reviewer comments should be present in full and each specific point should be responded to individually, point by point.

You should also cite any additional relevant literature that has been published since the original submission and mention any additional citations in your response.

2. In addition to a clean copy of the manuscript, please also upload a 'track-changes' version of your manuscript that specifies the edits made. This should be uploaded as a "Revised Article with Changes Highlighted " file type.

*Resubmission Checklist*

When you are ready to resubmit your revised manuscript, please refer to this resubmission checklist: https://plos.io/Biology_Checklist

To submit a revised version of your manuscript, please go to https://www.editorialmanager.com/pbiology/ and log in as an Author. Click the link labelled 'Submissions Needing Revision' where you will find your submission record.

Please make sure to read the following important policies and guidelines while preparing your revision:

*Published Peer Review*

Please note while forming your response, if your article is accepted, you may have the opportunity to make the peer review history publicly available. The record will include editor decision letters (with reviews) and your responses to reviewer comments. If eligible, we will contact you to opt in or out. Please see here for more details:

https://blogs.plos.org/plos/2019/05/plos-journals-now-open-for-published-peer-review/

*PLOS Data Policy*

Please note that as a condition of publication PLOS' data policy (http://journals.plos.org/plosbiology/s/data-availability) requires that you make available all data used to draw the conclusions arrived at in your manuscript. If you have not already done so, you must include any data used in your manuscript either in appropriate repositories, within the body of the manuscript, or as supporting information (N.B. this includes any numerical values that were used to generate graphs, histograms etc.). For an example see here: http://www.plosbiology.org/article/info%3Adoi%2F10.1371%2Fjournal.pbio.1001908#s5

*Blot and Gel Data Policy*

We require the original, uncropped and minimally adjusted images supporting all blot and gel results reported in an article's figures or Supporting Information files. We will require these files before a manuscript can be accepted so please prepare them now, if you have not already uploaded them. Please carefully read our guidelines for how to prepare and upload this data: https://journals.plos.org/plosbiology/s/figures#loc-blot-and-gel-reporting-requirements

*Protocols deposition*

To enhance the reproducibility of your results, we recommend that if applicable you deposit your laboratory protocols in protocols.io, where a protocol can be assigned its own identifier (DOI) such that it can be cited independently in the future. Additionally, PLOS ONE offers an option for publishing peer-reviewed Lab Protocol articles, which describe protocols hosted on protocols.io. Read more information on sharing protocols at https://plos.org/protocols?utm_medium=editorial-email&utm_source=authorletters&utm_campaign=protocols

Thank you again for your submission to our journal. We hope that our editorial process has been constructive thus far, and we welcome your feedback at any time. Please don't hesitate to contact us if you have any questions or comments.

Best regards,

Richard

Richard Hodge, PhD

Senior Editor, PLOS Biology

rhodge@plos.org

-----------------------------------------

REVIEWS:

Reviewer #1: This study provides a compelling investigation into the role of Ago2 in mediating resistance to immune checkpoint inhibitors (ICI) in non-small cell lung cancer (NSCLC), linking it mechanistically to interferon signaling suppression and reduced tumor immunogenicity. The findings are potentially significant and may provide new therapeutic avenues for converting immunologically 'cold' NSCLC tumors into 'hot' ones. However, several major concerns regarding the study's novelty, mechanistic depth, and conceptual clarity need to be adequately addressed before the manuscript can be considered for publication.

1. The authors state that "Ago2 is a driver of tumor growth" (line 145). If so, would overexpression of Ago2 be sufficient to promote tumor progression?

2. Given that Ago2 is a key player in the RNAi pathway, does its knockout (as shown in Figure 2) result in global dysregulation of gene expression across the genome—beyond the interferon signaling pathway?

3. Is the role of Ago2 in immunotherapy resistance solely through immune suppression, or does it have a dual function that also involves promoting tumor cell proliferation?

Specifically, did the authors compare the proliferation rates of Ago2-KO and wild-type (WT) cells in vitro? In Figure 3B, using Rag2−/−mice, Ago2 knockout and WT tumors exhibited comparable growth rates, suggesting that Ago2 loss may not significantly affect tumor growth in NSCLC. However, in hepatocellular carcinoma, Ago2 upregulation has been shown to enhance tumor cell proliferation, migration, and metastatic potential in vivo. How can these tissue-specific differences in Ago2 function be reconciled?

Furthermore, since treatment with ACF and ATA inhibitors suppresses tumor growth in Ago2-dependent models, is this effect solely due to off-target actions, or might Ago2 also directly influence tumor cell proliferation?

4. Wang et al. have recently reported that Ago2 mediates immunosuppression by inhibiting IFN-γ signaling, thereby contributing to immunotherapy failure. What novel mechanistic or conceptual insight does the present study provide regarding Ago2-mediated immune suppression?

5. The mechanism underlying Ago2-mediated immune suppression remains incompletely elucidated. The authors propose that Ago2 inhibits cytoplasmic dsRNA accumulation, thereby suppressing IFN signaling; however, what is the precise molecular mechanism involved? Additionally, while the model suggests that Ago2 suppresses TE transcription to inhibit ERVK and innate immune activation, Figures 4 and 5 focus on adaptive immunity. The direct relevance of these findings to the proposed innate immune pathway is not clearly established.

Reviewer #2: The manuscript titled "Ago2 loss restores sensitivity to immune checkpoint inhibitors in immunorefractory lung cancer" by Anobile et al. presents an interesting study investigating the role of Argonaute 2 (Ago2) in driving resistance to immune checkpoint inhibitor (ICI) therapy in non-small cell lung cancer (NSCLC). The authors utilize two well-established immunologically "cold" and ICI-resistant murine NSCLC models (LLC and KP). They demonstrate that genetic deletion of Ago2 in tumor cells significantly impairs tumor growth in immunocompetent mice but not in Rag2-deficient mice, highlighting the essential role of the adaptive immune system in this effect. Crucially, Ago2 loss sensitizes these otherwise refractory tumors to anti-PD-1 therapy, leading to markedly reduced tumor growth and even complete regressions in the KP model.

Mechanistically, the authors provide evidence that Ago2 deficiency leads to an upregulation of an interferon (IFN) signature and pro-inflammatory cytokines in tumors, both in vivo and in cell culture. They link this phenotype to the derepression of transposable elements (TEs) and a consequent accumulation of immunostimulatory double-stranded RNA (dsRNA) in Ago2-deficient cells. Flow cytometry analyses reveal that Ago2 loss, especially when combined with anti-PD-1, reshapes the tumor microenvironment, promoting the infiltration of immune cells, including CD8+ T cells, and fostering a more favorable T cell differentiation state. Finally, the clinical relevance is supported by analyzing an NSCLC patient cohort, showing that high tumor Ago2 expression correlates with poor survival after ICI treatment, while a high "Ago2-dependent" IFN gene signature correlates with improved survival.

Overall, this paper is well written, yet the relevance of the proposed mechanism is not sufficiently clear at this stage. There are a few major issues identified which are listed below that the authors should consider addressing:

1. In Figure1, authors adopted Ago2-knocked out cell lines. However, the current model does not rule out the possibility that Ago2 loss intrinsically impairs tumor cell growth, independent of the immune system. The use of Rag2⁻/⁻ mice, while informative, is not sufficient to dissect this potential confounder. To conclusively demonstrate that the restored ICI sensitivity is due to a reversible, immune-mediated mechanism, the authors should employ an inducible knockdown system (e.g., Doxycycline-inducible shRNA) to deplete Ago2 after tumors have been established.

2. The study lacks investigation into the therapeutic potential of combining an Ago2 inhibitor with anti-PD-1 antibody. To address this, experiments combining an Ago2 inhibitor with anti-PD-1 therapy should be performed in both the LLC and KP animal models to demonstrate synergistic efficacy.

3. In Figure4, authors demonstrated that knocking-out Ago2 in tumor cells influenced the immune infiltration in TDLNs. These findings are not fully compelling. To strengthen the study, the authors must address several key points:

1) Verification of TDLNs: They must provide experimental evidence, such as a dye injection assay, to conclusively demonstrate that the analyzed lymph nodes are indeed the tumor-draining lymph nodes (TDLNs).

2) Orthotopic Model Validation: The observations need to be validated in an orthotopic lung cancer model, as the tumor microenvironment and lymphatic drainage in a subcutaneous model may not faithful.

3) Molecular Mechanism: A more detailed molecular mechanism is needed to connect Ago2 loss to the observed immune activation. The proposed link involving transposable elements and dsRNA requires functional validation, as suggested in previous comments.

Reviewer #3: The present manuscript, titled "Ago2 loss restores sensitivity to immune checkpoint inhibitors in immunorefractory lung cancer," presents a significant finding. Genetic Ago2 deletion was observed to reduce tumor growth in immunocompetent animals. Notably, Ago2 deletion restored defective Type I interferon signaling and sensitized tumors to PD-1 immune checkpoint blockade.

The manuscript is logically structured, but it could benefit from linguistic improvements to enhance its readability.

Overall, the presented results are of great scientific interest and have already been validated by others, further substantiating the findings.

However, the translational value of the results is somewhat limited due to the off-target cytotoxicity exhibited by acriflavine in immunodeficient mice. It would have been beneficial to explore the use of aurintricarboxylic acid, which demonstrated less cytotoxic effects while simultaneously exhibiting tumor growth-inhibiting effects, even at low concentrations. Chemical inhibition of Ago2 (by potentially ATA, or BCI-137 as Wang et al. did) combined with PD-1 immune checkpoint blockade holds substantial translational promise.

# Author comment: Author response to Decision Letter 2

**DOI:** 10.1371/journal.pbio.3003860.r003

30 Apr 2026

Attachment Submitted filename: ResponseToReviewers_v5.pdf

# Peer review: Decision Letter 2

**DOI:** 10.1371/journal.pbio.3003860.r004

**Authors:** Richard Hodge

18 May 2026

Dear Dr Poirier,

Thank you for your patience while we considered your revised manuscript "Ago2 loss restores sensitivity to immune checkpoint inhibitors in immunorefractory lung cancer" for publication as a Short Report at PLOS Biology. This revised version of your manuscript has been evaluated by the PLOS Biology editors, the Academic Editor and the original reviewers.

Based on the reviews, I am pleased to say that we are likely to accept this manuscript for publication, provided you satisfactorily address the following data and other policy-related requests that I have provided below (A-J):

(A) We routinely suggest changes to titles to ensure maximum accessibility for a broad, non-specialist readership. In this case, we would suggest a minor edit to the title, as follows. Please ensure you change both the manuscript file and the online submission system, as they need to match for final acceptance:

"Argonaute 2 drives resistance to immune checkpoint inhibitors in immunorefractory non-small cell lung cancer"

(B) Your manuscript is being considered as a Short Report article type at the journal, which have a maximum of 4 main figures. At this stage, I would be grateful if you could please reduce the number of main figures to 4, either by moving one of the figures to the supplementary or by combining two of the main figures.

(C) In the animal ethics statement in the Methods section of the manuscript, please provide the specific approval number that was issued by the animal ethics committee to conduct the study. In addition, please confirm that an IACUC/animal ethics committee specifically reviewed and approved the mouse studies.

(D) You may be aware of the PLOS Data Policy, which requires that all data be made available without restriction: http://journals.plos.org/plosbiology/s/data-availability. For more information, please also see this editorial: http://dx.doi.org/10.1371/journal.pbio.1001797

Note that we do not require all raw data. Rather, we ask that all individual quantitative observations that underlie the data summarized in the figures and results of your paper be made available in one of the following forms:

-Supplementary files (e.g., excel). Please ensure that all data files are uploaded as 'Supporting Information' and are invariably referred to (in the manuscript, figure legends, and the Description field when uploading your files) using the following format verbatim: S1 Data, S2 Data, etc. Multiple panels of a single or even several figures can be included as multiple sheets in one excel file that is saved using exactly the following convention: S1_Data.xlsx (using an underscore).

-Deposition in a publicly available repository. Please also provide the accession code or a reviewer link so that we may view your data before publication.

Regardless of the method selected, please ensure that you provide the individual numerical values that underlie the summary data displayed in the following figure panels as they are essential for readers to assess your analysis and to reproduce it:

Fig. 1B-D; Fig. 2A-E; Fig. 3B-G; Fig. 4B-C; Fig. 5A, B; Fig. S1B-H; Fig. S2A-E; Fig. S3A-C, E; Fig. S4A-C; Fig. S5A, B; Fig. S7B; Fig. S8B and Fig. 9A, B

NOTE: the numerical data provided should include all replicates AND the way in which the plotted mean and errors were derived (it should not present only the mean/average values).

(E) Thank you for providing the RNA-sequencing data in the GEO database. However, we ask that the accession number of the deposition is now provided in the Data Availability Statement in the online submission form. In addition, please ensure that the data is made publicly available at this stage.

(F) For figures containing FACS data (Figures S7A and S8A) we ask that you provide FCS files and a picture showing the successive plots and gates that were applied to the FCS files to generate the figure. Please deposit this data in the FlowRepository (https://flowrepository.org/) or the Zenodo repository (https://zenodo.org/) and provide the accession number/URL of the deposition in the Data Availability Statement in the online submission form.

(G) Please also ensure that each of the relevant figure legends in your manuscript include information on *WHERE THE UNDERLYING DATA CAN BE FOUND*, and ensure your supplemental data file/s has a legend.

(H) We require the original, uncropped and minimally adjusted images supporting all blot and gel results reported in the following Figures:

Fig. S1A and S3D

We will require these files before a manuscript can be accepted so please prepare and upload them now. Please carefully read our guidelines for how to prepare and upload this data: https://journals.plos.org/plosbiology/s/figures#loc-blot-and-gel-reporting-requirements.

(I) Per journal policy, if you have generated any custom code during the course of this investigation, please make it available without restrictions. Please ensure that the code is sufficiently well documented and reusable, and that your Data Statement in the Editorial Manager submission system accurately describes where your code can be found. More information on our Code Policy, what and how to share can be found here: https://journals.plos.org/plosbiology/s/code-availability

Please note that we cannot accept sole deposition of code in GitHub, as this could be changed after publication. However, you can archive this version of your publicly available GitHub code to Zenodo. Once you do this, it will generate a DOI number, which you will need to provide in the Data Accessibility Statement (you are welcome to also provide the GitHub access information). See the process for doing this here: https://docs.github.com/en/repositories/archiving-a-github-repository/referencing-and-citing-content

(J) Please ensure that your Data Statement in the submission system accurately describes where your data can be found and is in final format, as it will be published as written there.

------------------------------------------------------------------------

As you address these items, please take this last chance to review your reference list to ensure that it is complete and correct. If you have cited papers that have been retracted, please include the rationale for doing so in the manuscript text, or remove these references and replace them with relevant current references. Any changes to the reference list should be mentioned in the cover letter that accompanies your revised manuscript.

In addition to these revisions, you may need to complete some formatting changes, which you will receive in a follow up email. A member of our team will be in touch with a set of requests shortly. If you do not receive a separate email within a few days, please assume that checks have been completed, and no additional changes are required.

We expect to receive your revised manuscript within two weeks.

To submit your revision, please go to https://www.editorialmanager.com/pbiology/ and log in as an Author. Click the link labelled 'Submissions Needing Revision' to find your submission record. Your revised submission must include the following:

- a cover letter that should detail your responses to any editorial requests, if applicable, and whether changes have been made to the reference list

- a Response to Reviewers file that provides a detailed response to the reviewers' comments (if applicable, if not applicable please do not delete your existing 'Response to Reviewers' file.)

- a track-changes file indicating any changes that you have made to the manuscript.

NOTE: If Supporting Information files are included with your article, note that these are not copyedited and will be published as they are submitted. Please ensure that these files are legible and of high quality (at least 300 dpi) in an easily accessible file format. For this reason, please be aware that any references listed in an SI file will not be indexed. For more information, see our Supporting Information guidelines:

https://journals.plos.org/plosbiology/s/supporting-information

*Published Peer Review History*

Please note that you may have the opportunity to make the peer review history publicly available. The record will include editor decision letters (with reviews) and your responses to reviewer comments. If eligible, we will contact you to opt in or out. Please see here for more details:

https://plos.org/published-peer-review-history/

*Press*

Should you, your institution's press office or the journal office choose to press release your paper, please ensure you have opted out of Early Article Posting on the submission form. We ask that you notify us as soon as possible if you or your institution is planning to press release the article.

*Protocols deposition*

To enhance the reproducibility of your results, we recommend that if applicable you deposit your laboratory protocols in protocols.io, where a protocol can be assigned its own identifier (DOI) such that it can be cited independently in the future. Additionally, PLOS ONE offers an option for publishing peer-reviewed Lab Protocol articles, which describe protocols hosted on protocols.io. Read more information on sharing protocols at https://plos.org/protocols?utm_medium=editorial-email&utm_source=authorletters&utm_campaign=protocols

Please do not hesitate to contact me should you have any questions.

Best regards,

Richard

Richard Hodge, PhD

Senior Editor, PLOS Biology

rhodge@plos.org

------------------------------------------------------------------------

Reviewer remarks:

Reviewer #1: I am satisfied with the author's revisions.

Reviewer #2: All of my previous concerns have been satisfactorily addressed. I have no additional comments at this stage.

Reviewer #3: The authors have thoroughly addressed the reviewers' comments and concerns in a comprehensive and satisfactory manner. In particular, they have provided additional in vivo experimental data that further substantiate the therapeutic potential of Ago2 inhibition in combination with immune checkpoint blockade in an orthotopic model of lung cancer. These experiments considerably strengthen the central conclusions of the study and provide important validation of the proposed therapeutic strategy in a physiologically relevant setting. The newly included data enhance mechanistic depth and improves the translational aspect of the work. The revised manuscript presents a clearer rationale for the combinatorial approach and more convincingly demonstrates its antitumor efficacy. Overall, the manuscript has been substantially improved and the work is now suitable for publication.

# Author comment: Author response to Decision Letter 3

**DOI:** 10.1371/journal.pbio.3003860.r005

4 Jun 2026

Attachment Submitted filename: ResponseToReviewers_v5_auresp_3.pdf

# Editor report: Decision Letter 3

**DOI:** 10.1371/journal.pbio.3003860.r006

**Authors:** Richard Hodge

4 Jun 2026

Dear Enzo,

On behalf of my colleagues and the Academic Editor, Hans-Uwe Simon, I am pleased to say that we can accept your manuscript for publication, provided you address any remaining formatting and reporting issues. These will be detailed in an email you should receive within 2-3 business days from our colleagues in the journal operations team; no action is required from you until then. Please note that we will not be able to formally accept your manuscript and schedule it for publication until you have completed any requested changes.

Please take a minute to log into Editorial Manager at http://www.editorialmanager.com/pbiology/, click the "Update My Information" link at the top of the page, and update your user information to ensure an efficient production process.

PRESS

We frequently collaborate with press offices. If your institution or institutions have a press office, please notify them about your upcoming paper at this point, to enable them to help maximise its impact. If the press office is planning to promote your findings, we would be grateful if they could coordinate with biologypress@plos.org. If you have previously opted in to the early version process, we ask that you notify us immediately of any press plans so that we may opt out on your behalf.

We also ask that you take this opportunity to read our Embargo Policy regarding the discussion, promotion and media coverage of work that is yet to be published by PLOS. As your manuscript is not yet published, it is bound by the conditions of our Embargo Policy. Please be aware that this policy is in place both to ensure that any press coverage of your article is fully substantiated and to provide a direct link between such coverage and the published work. For full details of our Embargo Policy, please visit http://www.plos.org/about/media-inquiries/embargo-policy/.

Thank you again for choosing PLOS Biology for publication and supporting Open Access publishing. We look forward to publishing your study.

Best regards,

Richard

Richard Hodge, PhD

Senior Editor, PLOS Biology

rhodge@plos.org

PLOS

Empowering researchers to transform science

Carlyle House, Carlyle Road, Cambridge, CB4 3DN, United Kingdom

California (U.S.) corporation #C2354500, based in San Francisco
